# Supplementary figures and images for: Surgical procedures for children in the public healthcare sector: a nationwide, facility-based study in Uganda
Source: BMJ Open. 2021 Jul 13;11(7):e048540. doi: 10.1136/bmjopen-2020-048540 (PMC8278888; doi:10.1136/bmjopen-2020-048540)

Appendix 1. Age distribution by hospital level.

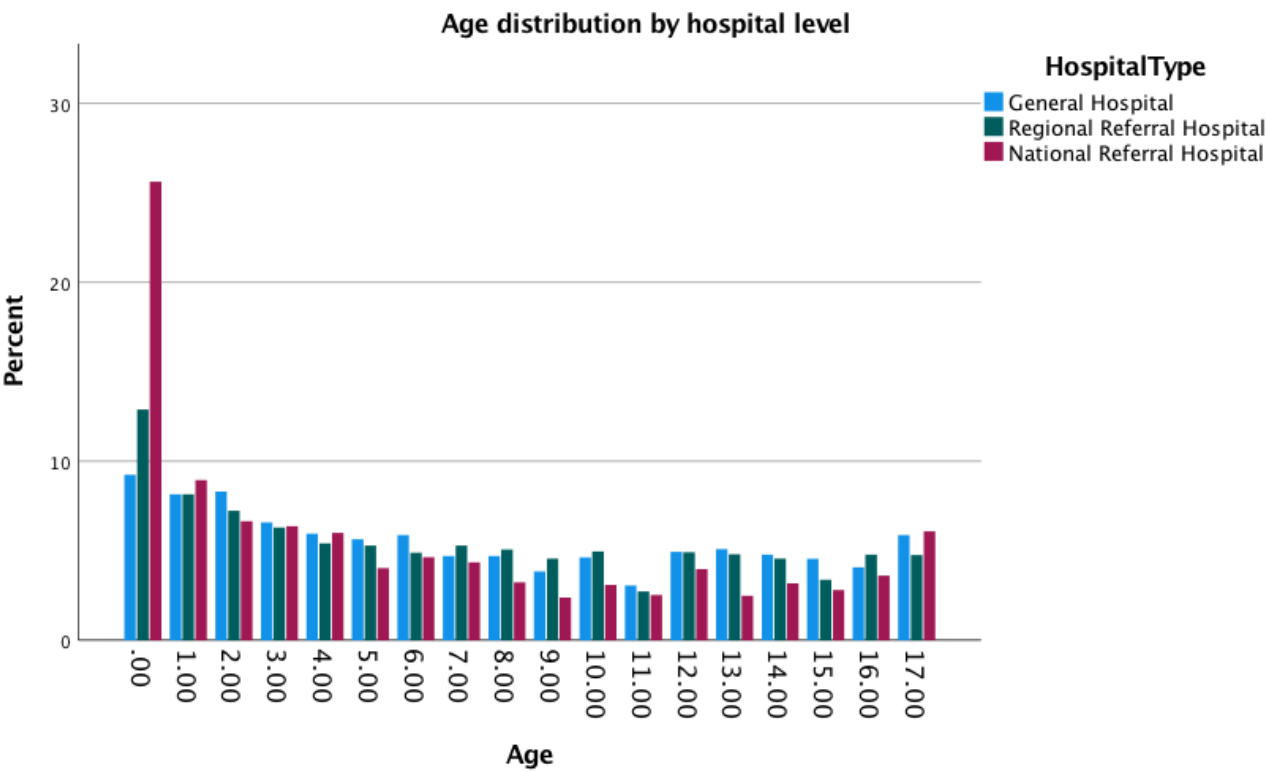

Supplement: Supplementary data [file bmjopen-2020-048540supp001.pdf]
