# Supplementary material for: Surgical procedures for children in the public healthcare sector: a nationwide, facility-based study in Uganda
Source: BMJ Open. 2021 Jul 13;11(7):e048540. doi: 10.1136/bmjopen-2020-048540 (PMC8278888; doi:10.1136/bmjopen-2020-048540)
Supplement: Author's manuscript [file bmjopen-2020-048540.draft_revisions.pdf]

BMJ Open is committed to open peer review. As part of this commitment we make the peer review history of every article we publish publicly available.

When an article is published we post the peer reviewers' comments and the authors' responses online. We also post the versions of the paper that were used during peer review. These are the versions that the peer review comments apply to.

The versions of the paper that follow are the versions that were submitted during the peer review process. They are not the versions of record or the final published versions. They should not be cited or distributed as the published version of this manuscript.

BMJ Open is an open access journal and the full, final, typeset and author-corrected version of record of the manuscript is available on our site with no access controls, subscription charges or pay-per-view fees (http://bmjopen.bmj.com).

If you have any questions on BMJ Open's open peer review process please email info.bmjopen@bmj.com

Surgery for children in the public health care sector in Uganda – a nationwide, facility-based study

Journal:	*BMJ Open*
Manuscript ID	bmjopen-2020-048540
Article Type:	Original research
Date Submitted by the Author:	30-Dec-2020

Complete List of Authors:	Ajiko, Mary Margaret; Soroti Regional Referral Hospital, Surgery<br>kressner, Julia; 1. Department of molecular medicine and Surgery, Karolinska Institute,<br>Matovu, Alphonsus; Mubende Regional Referral Hospital, Surgery; Karolinska Institute, Molecular Medicine and Surgery<br>Nordin, P.; Umeå University, Department of Surgical and Perioperative Sciences; Östersunds sjukhus, department of surgery<br>Wladis, Andreas; Linköping university, Department of Clinical and Experimental Medicine<br>Löfgren, Jenny; Karolinska Institute, Molecular Medicine and Surgery; Karolinska University Hospital, Reconstructive Plastic Surgery

Keywords:	SURGERY, PUBLIC HEALTH, PAEDIATRIC SURGERY

SCHOLARONE™
Manuscripts

I, the Submitting Author has the right to grant and does grant on behalf of all authors of the Work (as defined in the below author licence), an exclusive licence and/or a non-exclusive licence for contributions from authors who are: i) UK Crown employees; ii) where BMJ has agreed a CC-BY licence shall apply, and/or iii) in accordance with the terms applicable for US Federal Government officers or employees acting as part of their official duties; on a worldwide, perpetual, irrevocable, royalty-free basis to BMJ Publishing Group Ltd ("BMJ") its licensees and where the relevant Journal is co-owned by BMJ to the co-owners of the Journal, to publish the Work in this journal and any other BMJ products and to exploit all rights, as set out in our licence.

The Submitting Author accepts and understands that any supply made under these terms is made by BMJ to the Submitting Author unless you are acting as an employee on behalf of your employer or a postgraduate student of an affiliated institution which is paying any applicable article publishing charge ("APC") for Open Access articles. Where the Submitting Author wishes to make the Work available on an Open Access basis (and intends to pay the relevant APC), the terms of reuse of such Open Access shall be governed by a Creative Commons licence – details of these licences and which Creative Commons licence will apply to this Work are set out in our licence referred to above.

Other than as permitted in any relevant BMJ Author's Self Archiving Policies, I confirm this Work has not been accepted for publication elsewhere, is not being considered for publication elsewhere and does not duplicate material already published. I confirm all authors consent to publication of this Work and authorise the granting of this licence.

For peer review only

Surgery for children in the public health care sector in Uganda – a nationwide, facility-based study

Mary Margaret Ajiko (MD)[1,2], Julia Kressner (MD)[1], Alphonsus Matovu (MD)[3], Pär Nordin (PhD)[4], Andreas Wladis (PhD)[5], Jenny Löfgren (PhD)[1]

1. Department of Molecular Medicine and Surgery, Karolinska Institute, Sweden
2. Soroti Regional Referral Hospital, Uganda
3. Mubende Regional Referral Hospital, Uganda
4. Department of Surgery and Perioperative Sciences, Umeå University, Sweden
5. Department of Clinical and Experimental Medicine, Linköping University, Sweden

Corresponding author

Dr Jenny Löfgren,

Department of Molecular Medicine and Surgery, Karolinska Institutet.

Karolinska University Hospital L1:00. 17176 Stockholm, Sweden.

e-mail: jenny.lofgren@ki.se

Word Count: 3095

For peer review only - http://bmjopen.bmj.com/site/about/guidelines.xhtml

.

Abstract

Objective

This study investigated the surgical services for children at the highest levels of the public healthcare sector in Uganda. The aim was to determine volumes and types of procedure performed, the patients and the human resource involved.

Design

The study was a facility-based, record review.

Setting

The study was carried out at the National Referral Hospital, all 14 regional referral hospitals and 14 general hospitals in Uganda, representing the highest levels of hospital in the public healthcare sector.

Participants

The subjects were children <18 years who underwent major surgery in the study hospitals during 2013 and 2014.

Results

The study hospitals contribute with an average annual rate of paediatric surgery at 22.0 per 100,000 paediatric population. This is a fraction of the estimated need. Most of the procedures were performed for congenital anomalies (n=3111, 39.4%), inflammation and infection (n=2264, 28.7%), and trauma (n=1210, 15.3%). Specialist surgeons performed

For peer review only - http://bmjopen.bmj.com/site/about/guidelines.xhtml

60.3% (n=4758) of the procedures and anaesthesia was administered by specialist physician anaesthetists in 11.6% (n=917) of the cases.

Conclusions

A variety of paediatric surgical procedures are performed in a relatively decentralised system throughout Uganda. Task shifting and task sharing of surgery and anaesthesia are widespread: a large proportion of surgical procedures were carried out by non-specialist physicians, with anaesthesia mostly delivered by non-physician anaesthetists. Reinforcing the capacity and promoting the expansion of the health facilities studied, in particular the general hospitals and regional referral hospitals, could help reduce the immense unmet need for surgical services for children in Uganda.

For peer review only

For peer review only - http://bmjopen.bmj.com/site/about/guidelines.xhtml

Article summary

Strengths and limitations of this study

- This study set out to investigate surgical services for children at the highest levels of the public healthcare sector. Therefore, the national referral hospital, all 14 regional referral hospitals and 14 general (district) hospitals were included. Thus the internal validity is high.
- The study did not include lower-level hospitals or the private sector. For this reason, overall surgical rates for children cannot be calculated based on the study findings. This was, however, not the aim of the study.
- Due to the retrospective data collection, the level of detail is limited to that of the logbooks reviewed. Two study hospitals had to be excluded due to non-available or inadequate information. However, most operating theatre registers were available and as no national registers for surgery exist, the study provided important information about the state of surgery for children in Uganda.
- The data collected is now (2020) 6-7 years old, preceding recent global surgery efforts. A follow-up study is planned, for which the present results will serve as important baseline information for assessing whether / how far the global surgery movement has influenced surgical services for children in the public healthcare sector.

For peer review only - http://bmjopen.bmj.com/site/about/guidelines.xhtml

For peer review only

Introduction

In recent years, global surgery has become a field of its own with the goal of providing access to safe and affordable surgery for all. In 2017, 1.7 billion children and adolescents lacked access to surgical care (1). Many childhood surgical conditions can be treated successfully with surgery, while failure to do so may lead to lifelong complications, disability, or even death (2)(3). The provision of surgical services for children is highly cost-effective (4).

Children requiring surgical intervention make up a significant proportion of child admissions. Information available suggests that 6-12% of all paediatric admissions in sub-Saharan Africa are surgical, although the proportion may be higher in some urban areas (5). In a regional referral hospital in Uganda, a third of the patients admitted to a surgical department during a three-year period were below 18 years of age (6). This however is not enough to meet the need for surgical services for children. In a cross-sectional survey performed in Uganda, Sierra Leone, Rwanda and Nepal, 19% of children had a surgical condition and 62% of these children had at least one unmet surgical need (3). A study in Uganda found that 7.4% of children were currently suffering from an untreated surgical condition (7).

Uganda is a sub-Saharan, low-income country with a population of over 20 million children (8). With a birth rate of 5.0 children per women, the paediatric population is rapidly increasing (9). Although 80% of the Ugandan population live in rural areas, 95% of surgeons practice in an urban environment(10). The extent to which the healthcare system can provide surgical services for children at national level is not known. The aim of the study was to investigate volumes and types of paediatric surgery performed at the highest-level hospitals

For peer review only

in Uganda, to relate that to the need for surgery in the paediatric population and to describe the human resource delivering the services.

Method

Study design

This was a retrospective review of relevant hospital records.

Study location, study population and study size

Some 29 hospitals geographically distributed throughout Uganda were included. These hospitals represent the highest level of public health care and include the National Referral Hospital (Mulago), all 14 regional referral hospitals (RRH) and an additional 14 general hospitals (GH) (Figure 1). Data from two RRH were incomplete and they were therefore excluded from the analysis.

The public healthcare system in Uganda is divided into seven levels: from village health teams (level I) to health centres (levels II-IV) and hospitals (levels V-VII). The hospitals are divided into general (district) hospitals (GH, level V), regional referral hospitals (RRH, level VI) and the National Referral Hospital (NRH, level VII). All private hospitals, are classified as level V hospitals. Uganda has 113 general hospitals, 38 of which are public-service hospitals. The rest are private and private non-profit hospitals (11). Each RRH has at least one general hospital in its catchment area. Due to scarcity of suitable public general hospitals in the northern and eastern regions, two NGO general hospitals were included in the study.

For peer review only - http://bmjopen.bmj.com/site/about/guidelines.xhtml

The catchment populations between the GHs, RRHs and NRH overlap as they are meant to provide services of increasing complexity. The general hospitals and the regional referral hospitals were designed for a catchment population of 500,000 people and two million people respectively, but these hospitals cover much larger populations now due to rapid population growth. GHs have specialists in internal medicine, gynaecology and obstetrics and in paediatrics, but not in surgery. Non-specialist medical doctors perform surgical procedures. Each RRH has a mandate to receive referred patients from the regions they serve, but self-referral from other regions is also permitted. The RRHs employ general surgeons. The National Referral Hospital covers the entire population for highly specialised services. It is also a teaching facility of Makerere College of Health Sciences. The three paediatric surgeons and the paediatric anaesthetist in Uganda are employed at this institution.

Since 2001, all public healthcare facilities have been officially free of charge (12), which is why the public healthcare sector was selected for this survey. To describe the surgical activity for children here, the NRH and all the 14 RRHs were included. In addition, one general hospital per RRH was selected. It was hypothesized that the surgical activity for children in the GHs would be low, as no surgeons are employed in these hospitals. This selection of hospitals allowed comparisons between the three highest levels in the public health care sector.

Logbooks from operating theatres for the years 2013 and 2014 at the hospitals studied were photographed and the information extracted was entered into excel spreadsheets. Data on all major surgical procedures performed on children were included. The logbooks provided, for each hospital, information about children that had undergone a surgical procedure. They included date of procedure, age of the patient, diagnosis, type of surgical procedure

For peer review only - http://bmjopen.bmj.com/site/about/guidelines.xhtml

performed, names of surgeon and anaesthetist, and type of anaesthesia used. Each surgeon's and anaesthetist's training level was thereafter clarified with each hospital. In this study, a child is defined as "a human being below the age of 18 years", according to the United Nations Convention on Children's Rights. This is also in keeping with the Ugandan Constitution (13). In 2014, Uganda children 0-17 represented 55.1% of the population, or 19.1 million children (14).

A major surgical procedure was defined as "any procedure conducted in the operating theatre involving the incision, excision, manipulation or suturing of tissue which usually requires regional or general anaesthesia or profound sedation to control pain"(15). An anaesthetic assistant/officer was a healthcare provider who had undertaken a 2-3 year course in anaesthesia at a recognised institution, and had been certified by the Allied Health Professionals Council of Uganda (16). A senior house officer (SHO) was a resident in surgery or anaesthesia. An intern had completed medical school and formally practices under the supervision of a senior colleague. A specialist physician is a doctor who has completed advanced education and training in a specific field of medicine.

Data analysis

Data were analysed by region and by hospital type using standard descriptive methods. Data are presented to describe the basic characteristics of the patients, the procedures and the human resource providing the anaesthesia and surgical services. Analyses used for group comparisons were Pearson's *t* test for continuous data and the Chi square test for proportions and identification of associations between demographic characteristics and indications for surgery. We also categorised gender and age distribution with the number of procedures performed on children for each hospital level. Missing data on the number of procedures

For peer review only - http://bmjopen.bmj.com/site/about/guidelines.xhtml

performed was handled by multiple imputation. The surgical volumes at the NRH were distributed to the entire population with the relative proportion of 74% to the central region, 12% to the western region,13% to the eastern region and 1% to the northern region. This distribution was based on previous research on geographical distribution of paediatric surgical patients presenting to the NRH (17). The 2014 national population census for Uganda were used to calculate the rate of surgical procedures for children per 100,000 paediatric population for 2014. The paediatric population of Uganda for 2013 was calculated based on the 2014 national census and a population growth of 3.3% per year (18).

In order to relate the hospital surgical productivity to the need for surgery, a population-based study on paediatric surgical need carried out in Uganda in 2014 was used (7).

For identification of common diagnoses and procedures, only complete original data were used. Two regional referral hospitals were excluded from the analyses; one had no data for the entire study period and the other omitted patient age in the majority of cases, making it impossible to extract all paediatric cases.

Ethical considerations

Ethical approval was granted by the Makerere University School of Public Health (HDREC 076) and the Uganda National Council of Science and Technology (HS1892). The Medical Superintendent of each hospital approved collection of data from the theatre log books. The retrospective nature of the study meant that the treatment delivered to the patients was not affected. To protect their integrity, patients' names were not included.

For peer review only - http://bmjopen.bmj.com/site/about/guidelines.xhtml

Role of the funding source

The funder of the study had no role in the study design, data collection, data analysis, data interpretation, writing of the article or the decision to submit for publication. All authors had full access to all the data in the study and were responsible for the decision to submit for publication.

Patient and Public involvement

There was no involvement of patients or the public.

Results

A total of 7886 children who had undergone a major surgical procedure were identified in the theatre records of the 27 hospitals during the study period. After imputation for missing data, this number was 8238 of which 4680 were operated in 2013 and 3558 in 2014. This represented an average rate of 22.0 major surgical procedures per 100,000 paediatric population of Uganda in 2013-2014. The rate varied between the four regions in Uganda: from 12.0 per 100,000 children in the northern region to 27.7 per 100,000 children in the central region (Figure 1). There was considerable variation in surgical volumes within the RRH and GH groups and the most productive RRH performed 21 times more surgical procedures on children than the least productive.

A population-based study in Uganda previously investigated the prevalence of surgical conditions and the need for surgical consultation and surgical intervention among children in Uganda (7). In relation to the findings of this previous study, the study hospitals in the

For peer review only - http://bmjopen.bmj.com/site/about/guidelines.xhtml

present study contributed with surgical volumes corresponding to 4.9-7.6% of the need for major surgical procedures in children (Table 1).

The basic characteristics of the patients and the surgical and anaesthesia providers are shown in Table 2. Of the children, 43.4% (n=3422) were below five years of age, and 60.1% (n=4797) were boys. In Appendix 1, the age distribution by hospital level is visualised. Most of the procedures (n=4758, 60.3%) were performed by specialists in general surgery or specialists in other surgical specialities (paediatric surgery, gynaecology, orthopaedic surgery, cardiothoracic surgery, plastic surgery, neurosurgery, ear nose and throat surgery and urology). Overall, surgical residents performed 14.5% (n=1145) of the procedures and other non-specialist physicians performed 20.5% (n= 1616). Interns carried out 3.0% (n=237). Anaesthesia was rarely administered by a physician anaesthetist (n=917, 11.6%) or physician anaesthetic resident (n=818, 10.4%). The majority of the patients were anaesthetised by an anaesthetic assistant or anaesthetic officer (n=5469, 69.4%).

The surgical procedures were performed by 275 medical doctors with different levels of qualification: 103 specialist surgeons in different disciplines of whom three were paediatric surgeons, 57 resident surgeons in different surgical disciplines and 115 medical officers without specific training in surgery. Of the specialists, 40 performed surgery at the NRH and 61 at the RRH. Only two specialist surgeons performed surgical procedures at a GH. Anaesthesia was administered by 18 physician anaesthetists and 99 anaesthetic officers. Of the physician anaesthetists, three worked at a RRH and 15 at the NRH. No physician anaesthetist worked at a GH.

For peer review only - http://bmjopen.bmj.com/site/about/guidelines.xhtml

Indications for the surgical procedures are presented in Table 2. The most common were related to congenital anomalies (n=3111, 39.4%) infectious and inflammatory conditions (n=2264, 28.7%) and trauma (n=1210, 15.3%). Table 3 shows the surgical procedures that were carried out. Abdominal and colorectal surgical procedures were most common, followed by orthopaedic and ear, nose and throat (ENT) procedures. The types of procedure varied, and in the GH hernia surgery was the most common. Surgery for congenital heart conditions (n=128, 1.6%) was only performed at the NRH where there is a heart institute run by cardiothoracic surgeons. Neurosurgeons performed all neurosurgical procedures including ventriculoperitoneal shunts, spina bifida repair, brain abscess drainage, craniotomy and meningocele repair. Such procedures were carried out at the NRH (Mulago Hospital) and some RRHs.

Discussion

The study hospitals in the present study contributed with an average annual surgical rate of 22.0 major surgical procedures per 100,000 paediatric population in Uganda in 2013 and 2014. The patients were often below five years and the surgical procedures were mainly carried out for congenital anomalies, inflammatory and infectious conditions and trauma. Task sharing and task shifting were widely practised as non-specialists frequently performed surgical procedures and as non-physician clinicians administered anaesthesia in most of the cases.

A considerable variation in the number and rate of major procedures performed at the different hospitals, and within the four regions of the country, was seen. The northern region

For peer review only - http://bmjopen.bmj.com/site/about/guidelines.xhtml

was the most underserved, where hospitals are few and the distance to the national referral hospital is greatest. This is similar to the results of a previous study in Uganda, which found a positive correlation between burden of paediatric surgical disease and distance to the nearest healthcare unit offering surgery for children (19). The majority of the Ugandan population live in a rural setting with very limited financial resources (6)(20). Transport costs are likely to pose a barrier to surgical care for children there. This is confirmed by previous research in low- and middle-income countries showing that cost and travelling distance are barriers to surgical care access (21). Decentralised services are therefore needed.

The Global Initiative for Children's surgery developed the Optimal Resources for Children's Surgical Care, published in 2019. This document stratifies by level of hospital what services and human resource should be available at each level. The recommendations are in line with what is demonstrated in the present study. On GH level, intermediate surgical services, including hernia repair and acute abdominal and traumatic conditions, should be performed (22). In the general hospitals in the present study, hernia surgery represented the vast majority of procedures performed, followed by other abdominal and orthopaedic procedures. These procedures were carried out on all three levels but in addition, the RRHs and NRH performed procedures of increasing complexity including surgical subspecialities.

For essential surgical care to be accessible for all children in Uganda, service delivery in both GHs and RRHs should be further promoted. It is encouraging to see that surgery for children was delivered in these hospitals even if at rates that represent only a fraction of the need. The more productive hospitals in each group could serve as models for the less productive ones. A first goal would be for all hospitals on the same level to deliver the same services and similar volumes of surgery. The present study did not investigate the outcomes after surgery,

For peer review only - http://bmjopen.bmj.com/site/about/guidelines.xhtml

and this should be evaluated in future research. Volume and quality represent two sides of the same coin and capacity-building must take both into consideration.

Significantly more boys than girls were recorded in this study. Several of the most common diagnoses requiring surgery were gender-specific, or over-represented, in males. For example, inguinal hernia, which was the most common indication for surgery in the GHs, is more common in boys than in girls (23). Female-specific diagnoses such as gynaecological conditions were rare. This study could not determine whether boys have better access to surgery than girls, but future research into gender as a barrier to surgical care in children could investigate this further.

Task-sharing and task-shifting of surgical duties and anaesthesia delivery were routinely practiced in the study hospitals. Task sharing with medical doctors without specific training in surgery is common in many countries but the safety and effectiveness as well as the training available for this important group of surgical providers is not well understood (24). Ways in which the highest possible quality of paediatric surgical services can be achieved and maintained must be the subject of future research. Standardization of the surgical training, and support and monitoring of non-surgeons for the commonest surgical conditions should be prioritized. The anaesthetic officers in Uganda have formal training and their duties are probably better regulated than those of non-specialist medical doctors. Nonetheless, regular monitoring of the surgical and anaesthetic work forces and the institutions in which they work is an essential component of the provision of high-quality and cost-effective surgical services for children. An additional important role of the health care system is to promote prevention: many surgical conditions can be prevented. Antenatal care and

For peer review only - http://bmjopen.bmj.com/site/about/guidelines.xhtml

vaccinations are carried out in the study hospitals and health information and child safety issues could be discussed with parents during such visits.

Surgery has an important place in several of the health targets in the UN's Sustainable Development Goal 3 (25). Uganda has a national plan for service standards, and service delivery standards for the health sector include a package for surgical, obstetrics, anaesthesia plans in scaling up surgical volumes and access to surgical care. However, a defined paediatric surgical package is not included (26). To reduce the death toll and disability caused by surgical conditions in the paediatric population, the healthcare system must be improved in order to deliver safe surgery and prevention strategies. Advancing and promoting the service delivery already in place could be part of the solution to reach the goal of surgical services of quality for all children with surgical conditions.

Strengths and Limitations

This study set out to investigate the situation for surgery for children in the public healthcare system in Uganda and to describe patients and their surgical providers at the three highest levels of hospitals. The study has several limitations. Due to limited resources, not all hospitals in the country could be visited and therefore overall volumes and surgical rates could not be calculated. The hospitals studied were the healthcare facilities first in line for the implementation of new policies of intervention, where Ministry of Health policy as well as increased funding would have a direct effect. Our choice of hospitals was thus relevant regarding current capacity and surgical volume, providing baseline information that is essential in the future evaluation of the effects of investment and interventions made to enhance and expand paediatric surgical services.

For peer review only - http://bmjopen.bmj.com/site/about/guidelines.xhtml

Given the retrospective collection of data in this study, the level of detail is limited to that of the logbooks reviewed. Information on outcome after paediatric surgery in relation to level of hospital and level of training of the surgical and anaesthesia providers would be very valuable. Where data on volumes of surgery performed during the study period were missing, in the selected hospitals, these were imputed as the best option. However, the data sufficed to provide important information about the state of surgery for children in Uganda.

The present data were collected five years ago and since then no significant investments have been made for improving surgical services for children on national level. There is still (2020) only one paediatric anaesthetist, three registered paediatric surgeons, and two hospitals with dedicated paediatric operating theatres. The findings should thus still apply today.

Acknowledgements

The authors acknowledge the invaluable assistance of the hospital directors and staff which made the study possible. The authors would like to thank Anna Peterson, William Yu and Andreas Vikholm for their important contribution in data collection and management. We also thank Dr Matteo Bottai for statistical assistance.

Funding

This work was supported through a grant from the Department of Public Health and Clinical Medicine, Unit of Research, Education, and Development, Östersund, Umeå University.

Data sharing

Data may be shared upon request to the corresponding author.

For peer review only - http://bmjopen.bmj.com/site/about/guidelines.xhtml

Conflict of competing interests

There are no personal conflicts of interest or financial conflicts of interest among the authors.

Authors' Contributions

Study conception and design:
Jenny Löfgren, Andreas Wladis, Ajiko Mary Margaret, Pär Nordin.

Data acquisition:
Ajiko Mary Margaret, Jenny Löfgren, Julia Kressner, Alphonsus Matovu

Analysis and data interpretation:
Ajiko Margaret Ajiko, Jenny Löfgren, Julia Kressner, Andreas Wladis, Pär Nordin, Alphonsus Matovu

Drafting of the manuscript:
Ajiko Mary Margaret

Critical revision,
Ajiko Mary Margaret, Jenny Löfgren, Andreas Wladis, Par Nordin, Alphonsus matovu, Julia Kressner.

References

1. Mullapudi B, Grabski D, Ameh E, Ozgediz D, Thangarajah H, Kling K, et al. Estimates of number of children and adolescents without access to surgical care. Bull World Health Organ. 2019 Apr 1;97(4):254–8.

2. Walker IA, Obua AD, Mouton F, Ttendo S, Wilson IH. Paediatric surgery and anaesthesia in south-western Uganda: a cross-sectional survey. Bull World Health Organ. 2010 Dec 1;88(12):897–906.

3. Bickler SW, Rode H. Surgical services for children in developing countries. Bull World Health Organ. 2002;80:829–35.

4. Saxton AT, Poenaru D, Ozgediz D, Ameh EA, Farmer D, Smith ER, et al. Economic Analysis of Children's Surgical Care in Low- and Middle-Income Countries: A Systematic Review and Analysis. PloS One. 2016;11(10):e0165480.

For peer review only - http://bmjopen.bmj.com/site/about/guidelines.xhtml

5. Linden AF, Sekidde FS, Galukande M, Knowlton LM, Chackungal S, McQueen KAK. Challenges of surgery in developing countries: a survey of surgical and anesthesia capacity in Uganda's public hospitals. World J Surg. 2012 May;36(5):1056–65.

6. Tumusiime G, Was A, Preston MA, Riesel JN, Ttendo SS, Firth PG. The Quality and Utility of Surgical and Anesthetic Data at a Ugandan Regional Referral Hospital. World J Surg. 2017 Feb;41(2):370–9.

7. Butler EK, Tran TM, Fuller AT, Brammell A, Vissoci JR, de Andrade L, et al. Quantifying the pediatric surgical need in Uganda: results of a nationwide cross-sectional, household survey. Pediatr Surg Int. 2016;32(11):1075–85.

8. Population ages 0-14, total - Uganda | Data [Internet]. [cited 2020 Dec 22]. Available from: https://data.worldbank.org/indicator/SP.POP.0014.TO?locations=UG

9. Fertility rate, total (births per woman) - Uganda | Data [Internet]. [cited 2020 Dec 22]. Available from: https://data.worldbank.org/indicator/SP.DYN.TFRT.IN?locations=UG

10. Andersson O, Radell P, Ringo V, Mulungu M, Baker T. Quality of pediatric anesthesia: A cross-sectional study of a university hospital in a low-income country. PloS One. 2018;13(4):e0194622.

11. Kisa P, Grabski DF, Ozgediz D, Ajiko M, Aspide R, Baird R, et al. Unifying children's surgery and anesthesia stakeholders across institutions and clinical disciplines: challenges and solutions from Uganda. World J Surg. 2019;1–15.

12. Uganda Ministry of Health. Health Sector Strategic Plan 2000/01 - 2004/05 [Internet]. Available from: https://afri-can.org/wp-content/uploads/2016/04/HSSP-2000-01.pdf

13. Children Act. In: Ugandan Constitution. 1997.

14. Uganda Bureau of Statistics. National Population and Housing Census 2014 - Main Report [Internet]. [cited 2018 Jun 9]. Available from: http://www.ubos.org/onlinefiles/uploads/ubos/NPHC/NPHC%202014%20FINAL%20RESULTS%20REPORT.pdf

15. World Health Organization. Safe surgery saves lives. In: Second global patient safety challenge. 2008. p. 4.

16. Allied Health Professionals Council. The Allied Health Professionals Act - Chapter 268. In: The Allied Health Professionals Act.

17. Ullrich SJ, Kakembo N, Grabski DF, Cheung M, Kisa P, Nabukenya M, et al. Burden and Outcomes of Neonatal Surgery in Uganda: Results of a Five-Year Prospective Study. J Surg Res. 2020;246:93–9.

18. Population growth (annual %) - Uganda | Data [Internet]. [cited 2020 Dec 22]. Available from: https://data.worldbank.org/indicator/SP.POP.GROW?locations=UG

For peer review only - http://bmjopen.bmj.com/site/about/guidelines.xhtml

For peer review only

19. Smith ER, Vissoci JRN, Rocha TAH, Tran TM, Fuller AT, Butler EK, et al. Geospatial analysis of unmet pediatric surgical need in Uganda. J Pediatr Surg. 2017;52(10):1691–8.

20. Poverty headcount ratio at USD3.10 a day (2011 PPP) (% of population) [Internet]. The World Bank; 2012. Available from: data.worldbank.org/indicator/SI.POV.NAHC?locations=UG

21. Grimes CE, Bowman KG, Dodgion CM, Lavy CBD. Systematic Review of Barriers to Surgical Care in Low-Income and Middle-Income Countries. World J Surg. 2011 May;35(5):941–50.

22. Optimal Resources for Children's surgery [Internet]. Global Initiative for Children's Surgery. [cited 2020 Dec 6]. Available from: https://www.globalchildrenssurgery.org/optimal-resources/

23. Burcharth J, Pedersen M, Bisgaard T, Pedersen C, Rosenberg J. Nationwide Prevalence of Groin Hernia Repair. PLOS ONE. 2013 Jan 14;8(1):e54367.

24. Falk R, Taylor R, Kornelsen J, Virk R. Surgical Task-Sharing to Non-specialist Physicians in Low-Resource Settings Globally: A Systematic Review of the Literature. World J Surg. 2020 May;44(5):1368–86.

25. Home — SDG Indicators [Internet]. [cited 2020 Aug 19]. Available from: https://unstats.un.org/sdgs/#

26. HEALTH SECTOR DEVELOPMENT PLAN 2015/16 - 2019/20. :110.

For peer review only

Table 1. Surgical need and contribution towards the met need by the study hospitals

	Need for surgical consultation	Need for surgical intervention	Children operated in study hospitals	Interpretation
**Proportion of children**	7.4% *	7.6% - 11.8% of children with need for surgical consultation *	4.9 - 7.6 % of estimated need for a major surgical procedure	The 8238 children operated correspond to 4.9-7.6% of the estimated need for a major surgical procedure in the paediatric population of Uganda in 2014
**Number of children**	1,413,400 * <br>(7.4% of 19.1 million paediatric population in 2014)	107,418 – 166,781 *	8238	Another 99,000 - 159,000 surgical procedures are needed to meet the need for major surgical interventions in children in Uganda
**Rate, n per 100,000 paediatric population**	7400 *	562 – 873 *	22.0	The surgical rate contributed by the study hospitals is a fraction of the estimated need.

*** Reference 7. Butler et al**

For peer review only

Table 2. Basic characteristics of patients and human resource performing surgery for children per hospital type

	General hospital, n=1276	Regional Referral Hospital, n=4475	National Referral Hospital, n=2135	Total n=7886	p-value
**Sex, n (%)**					
Male	945 (74.1)	2516 (56.2)	1336 (62.6)	4797 (60.8)	<0.001*
Female	324 (25.4)	1620 (36.2)	756 (35.4)	2700 (34.2)	
Not indicated	7 (0.5)	339 (7.6)	43 (2)	389 (5.0)	
**Age distribution (years)**					
Mean (SD)	7.4 (5.5)	7.1 (5.5)	5.8 (5.7)	5.3 (4.7)	<0.001*
<5	488 (38.2)	1790 (40.0)	1144 (53.6)	3422 (43.4)	
>5	777 (60.9)	2677 (59.8)	980 (45.9)	4434 (56.6)	
Not indicated	11 (0.9)	8 (0.2)	11 (0.5)	30 (0.4)	
**Level of training of surgical provider**					
Specialist surgeon (including 3 paediatric surgeons)	324 (25.4)	3063 (68.4)	1371 (64.2)	4758 (60.2)	<0.001*
Resident	0	437 (9.8)	708 (33.2)	1145 (14.5)	
Medical officer	904 (70.8)	700 (15.6)	12 (0.6)	1616 (20.5)	
Intern	0	232 (5.2)	5 (0.2)	237 (3.0)	
Not indicated	48 (3.8)	43 (1.0)	39 (1.8)	130 (1.6)	
**Level of training of Anaesthesia provider**					
Physician Anaesthetists, (one paediatric anaesthesiologist)	0	807 (18.0)	110 (12.0)	917 (11.6)	<0.001*
Residents in anaesthesia,	0	163 (3.6)	655 (30.7)	818 (10.4)	
Self/by surgeon local anaesthesia	47 (3.7)	162 (3.6)	10 (0.5)	219 (2.8)	
Anaesthetic assistant or Officer	1190 (93.3)	3132 (70)	1147(53.7)	5469 (69.4)	
Not indicated	39 (3.1)	211 (4.7)	213 (10)	463 (5.9)	
**Type of Anaesthesia**					
General anaesthesia	1097 (86.0)	3811 (85.2)	1913 (89.6)	6821 (86.5)	<0.001*
Spinal anaesthesia	66 (5.2)	186 (4.2)	82 (3.8)	334 (4.2)	
Local anaesthesia ± sedation	106 (8.3)	161 (3.6)	64 (3.0)	331 (4.2)	
Not indicated	7 (0.5)	317 (7.1)	76 (3.6))	400 (5.1)	

* Represents p-values of comparison of gender, age, level of training of surgical and anaesthesia provider and type of anaesthesia between GH, RRH and the NRH respectively.

For peer review only

Table 3. Indications for surgical procedures for children in the public healthcare sector in Uganda 2013-2014

	General Hospital 1276 patients, n (%)	Regional Referral Hospital 4475 patients, n (%)	National Referral Hospital 2135 patients, n (%)	Total 7886 patients, n (%)
**Abdominal and colorectal symptoms and conditions (n= 3662)**				
Groin hernia and hydrocoele	499 (39.1)	650 (14.5)	120 (5.6)	1269 (16.1)
Peritonitis	49 (3.8)	318 (7.1)	106 (5.0)	473 (6.0)
Umbilical hernia	113 (8.9)	185 (4.1)	72 (3.4)	370 (4.7)
Gut perforation	31 (2.4)	240 (5.4)	61 (2.9)	332 (4.2)
Imperforate anus	13 (1.0)	109 (2.4)	163 (7.6)	285 (3.6)
Intussusception	18 (1.4)	130 (2.9)	126 (5.9)	274 (3.5)
Intestinal Obstruction	47 (3.7)	124 (2.8)	89 (4.2)	260 (3.2)
Appendicitis	24 (1.9)	60 (1.3)	42 (2.0)	126 (1.6)
Abdominal injuries (blunt & penetrating)	10 (0.8)	62 (1.4)	37 (1.7)	109 (1.4)
Ruptured spleen	12 (0.9)	60 (1.3)	18 (0.8)	90 (1.1)
Colostomy/ closure of colostomy	10 (0.8)	44 (1.0)	20 (0.9)	74 (1.0)
**Orthopaedic conditions (n=1264)**				
Orthopaedic trauma	62 (4.9)	366 (8.2)	224 (10.5)	652 (8.2)
Chronic osteomyelitis and septic arthritis	137 (10.7)	254 (5.7)	82 (3.8)	473 (6.0)
Club foot	61 (4.8)	53 (1.2)	25 (1.2)	139 (1.8)
Total	260 (20.4)	673 (15.0)	331 (15.5)	1264 (16.0)
**Ear, Nose, Throat conditions (n=718)**				
Tonsillitis/enlarged adenoids/ Foreign body	0 (0)	547 (12.2)	171 (8.0)	718 (9.1)
**Urology (n=441)**				
Cryptochidism	30 (2.4)	63 (1.4)	12 (0.6)	105 (1.3)
Testicular torsion	8 (0.6)	41 (0.9)	36 (1.7)	85 (1.1)
Other	6 (0.5)	138 (3.1)	107 (5.0)	251 (3.2)
**Neurosurgical conditions (n=316)**				
Spina bifida, hydrocephalus, Meningocoele, subdural haemorrhage, trauma	0 (0.0)	135 (3.0)	181 (8.5)	316 (4.0)
**Congenital heart disease (n=125)**	0 (0.0)	3 (0.1)	122 (5.7)	125 (1.6)
**Plastic surgical conditions (n=503)**				
Cleft lip and palate	27 (2.1)	107 (2.4)	10 (0.5)	144 (1.8)
Acute burns and contractures	50 (3.9)	243 (5.4)	66 (3.1)	359 (4.6)
**Malignancies (n=92)**	1 (0.1)	68 (1.5)	24 (1.1)	92 (1.1)
**Other*, (n=1008) goitre, bladder calculi, liver disease, burst abdomen, big spleen disease, cataract, foreign body in eye, pyloric stenosis**	90 (7.0)	705 (15.8)	213 (10.0)	1008 (12.8)
**Not indicated (n=51)**	7 (0.5)	36 (0.8)	8 (0.4)	51 (0.6)

* Indications with ≤ 1%, were grouped together under "other". Some patients had more than one indication for surgery, hence total number of conditions and symptoms exceeds number of patients.

For peer review only - http://bmjopen.bmj.com/site/about/guidelines.xhtml

Table 4. Paediatric surgical procedures by hospital level in Uganda 2013-2014.

Procedure	General Hospital, 1276 patients, n (%)	Regional Referral Hospital, 4475 patients, n (%)	National Referral Hospital, 2135 patients, n (%)	Total n=7886 patients, n (%)
**Abdominal and colorectal surgery (n=4559 procedures)**				
Laparotomy	179 (14.0)	990 (22.1)	453 (21.2)	1622 (20.6)
Herniotomy/herniorrhaphy/hydrocelectomy	591 (46.3)	652 (14.6)	121 (5.7)	1364 (17.3)
Colostomy (creating/closure)	17 (1.3)	206 (4.6)	175 (8.2)	398 (5.0)
Mayo's repair of umbilical hernia	113 (8.9)	179 (4.0)	68 (3.2)	360 (4.6)
Resection and anastomosis	12 (0.9)	149 (3.3)	83 (3.9)	244 (3.1)
Appendicectomy	30 (2.4)	58 (0.8)	45 (2.1)	133 (1.7)
Splenectomy	7 (0.5)	73 (1.6)	13 (0.6)	93 (1.2)
Other abdominal procedures (Swenson pull through, posterior sagittal anorectoplasty, Ramtsaedt's procedure.)	0 (0.0)	195 (4.4)	150 (7.0)	345 (4.4)
**Orthopaedic procedures (n=1212 procedures)**				
Fracture surgery (internal and external fixations), amputations.	72 (5.6)	452 (10.1)	154 (7.2)	678 (8.6)
Sequestrectomy	115 (9.0)	141 (3.2)	54 (2.5)	310 (3.9)
Corrective procedures for club foot	61 (4.8)	63 (1.4)	25 (1.2)	149 (1.9)
Arthrotomy	9 (0.7)	43 (1.0)	23 (1.1)	75 (1.0)
**Ear Nose Throat (n=594 procedures)**				
Adenotonsillectomy	0 (0)	292 (6.5)	115 (5.4)	407 (5.2)
FB removal, bronchoscopy, others	2 (0.2)	134 (3.0)	51 (2.4)	187 (2.4)
**Urology (n=429)**				
Orchidopexy and orchidectomy	41 (3.2)	115 (2.6)	34 (1.6)	190 (2.4)
Reconstructive surgery (hypospadia, epispadia, urethral valve)	4 (0.3)	130 (3.0)	105 (5.0)	239 (3.0)
**Cardiothoracic and vascular surgery (n=128 procedures)**	0 (0)	0 (0)	128 (6.0)	128 (1.6)
**Plastic Surgery (n=293 procedures)**				
Cleft lip and palate	31 (2.4)	132 (2.9)	50 (2.3)	213 (2.7)
Skin grafting	22 (1.7)	43 (1.0)	15 (0.7)	80 (1.0)
**Neurosurgery (n=285 procedures)**				
Neurosurgery procedures (VP shunt, Drainage of brain abscess, craniotomy, Repair of meninges	0 (0)	122 (2.7)	163 (7.6)	285 (3.6)
**Other procedures (n=782 procedures)***	92 (7.2)	575 (12.8)	115 (5.4)	782 (9.9)
**Not indicated (n=16 procedures)**	0 (0)	16 (0.4)	0 (0)	16 (0.2)

* Procedures with ≤ 1%, were grouped under "other". Some patients had more than one surgical procedure performed, hence total number of procedures exceeds number of patients.

For peer review only - http://bmjopen.bmj.com/site/about/guidelines.xhtml

Figure 1. Volumes and rates of paediatric surgery per region in Uganda.

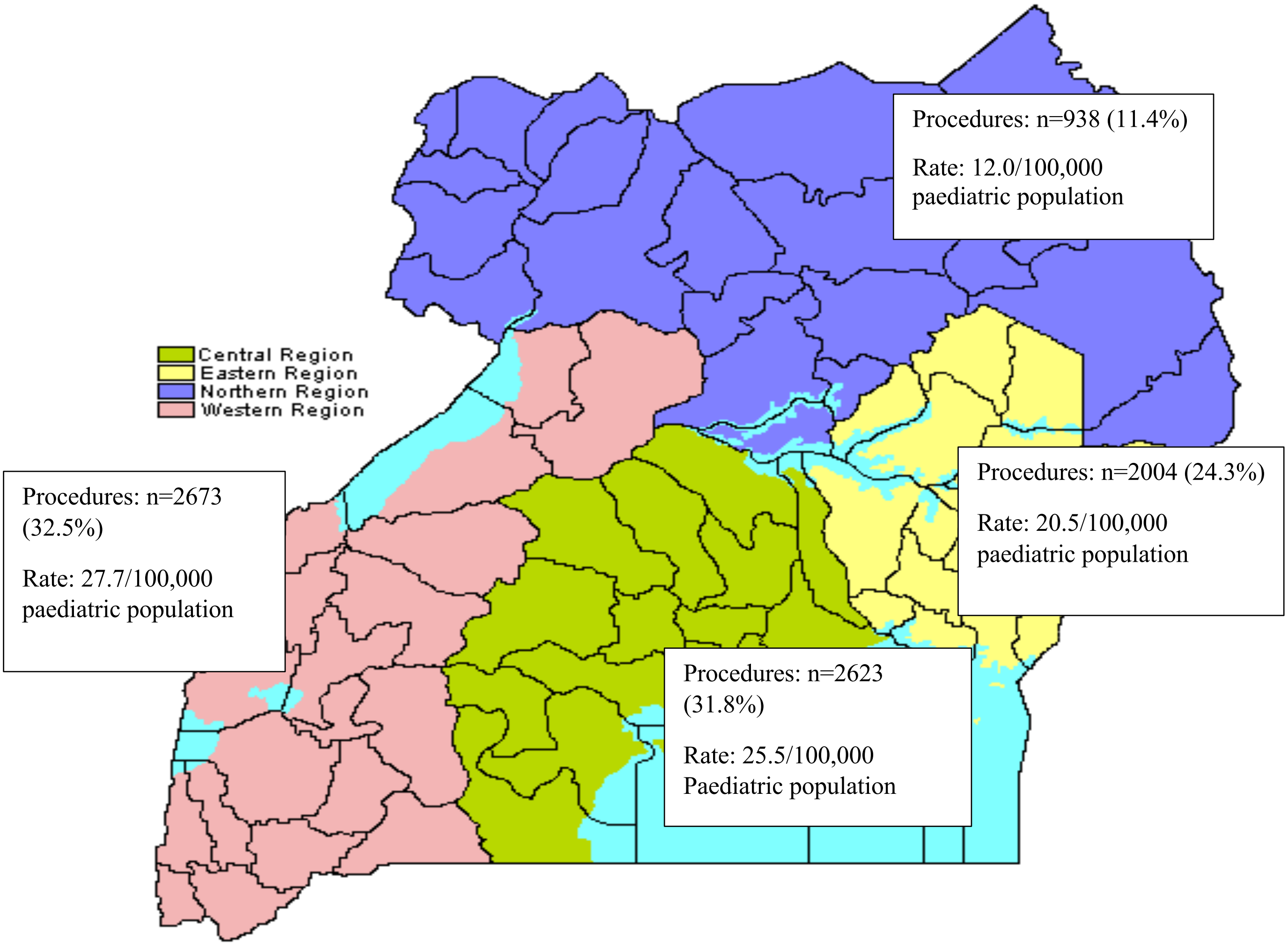

Northern region: 7,188,139 inhabitants, (Apac GH, Nebbi GH, Kitgum GH, Matany GH, Lira RRH, Gulu RRH, and Moroto RRH). **Eastern region**: 9,042,422 inhabitants, (Lwala GH, Tororo GH, Iganga GH, Soroti RRH, Mbale RRH, Jinja RRH). **Western region**: 8,874,862 inhabitants, (Kilembe GH, Kisoro GH, Masindi GH, Itojo GH, Hoima RRH, Fort portal RRH, Mbarara RRH/University teaching hospital. **Central region**: 9,529,227 inhabitants. (Entebbe GH, Kalizizo GH, Mityana GH, Masaka RRH, Naguru RRH, Mubende RRH, Mulago NRH/Makerere University teaching hospital).

Uganda Bureau of Statistics. National Population and Housing Census 2014. https://www.ubos.org/wp-content/uploads/publications/03_20182014_National_Census_Main_Report.pdf.Total pop 34.6 million 0-17 yrs.=55.1%

Appendix 1. Age distribution by hospital level.

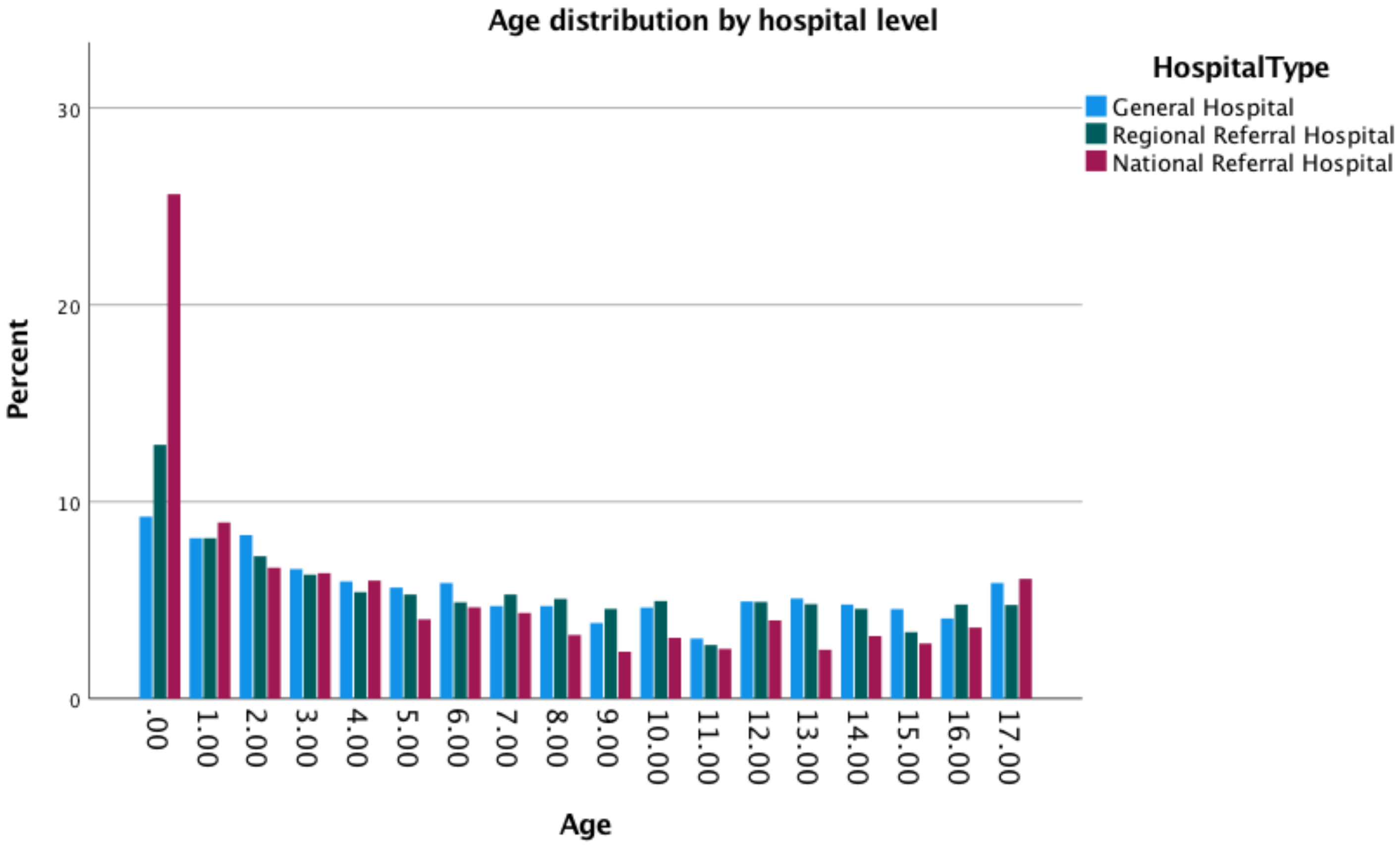

review only

Responses to reviewers' comments

Dear Editor,

Thank you for considering this manuscript for publication. We also want to thank the reviewers for their detailed and very valuable comments and recommendations. We have made extensive updates to the manuscript including updated statistical analyses. Below, we have made a point-by-point response to the reviewers comments.

Reviewer: 1

Specific study report.
Page 5, line 31: how can you make this statement? In the results later on you do not really divide surgery in emergency vs elective for each condition so the reader does not really know how many hernias were done as electives and how many as incarcerated so this statement might be true but cannot be extrapolated from the data you are presenting.

Thank you for this observation. You are correct that the data could not support to which extent that some procedures were carried out as emergencies or as elective procedures. Hernia is a typical such condition where it would have been valuable to know the distribution of emergency and elective surgical care. However, a number of the indications for surgery are emergent. Examples that are always treated as emergencies are peritonitis, appendicitis, gut perforation, intestinal obstruction, ruptured spleen, orthopaedic trauma, to name a few. The conditions that always require emergency surgical care together represent almost 40%, and this is a significant proportion of the total.

Introduction:
Page 5 line 40, the first sentence does not make much sense. Perhaps you can delete it and incorporate it in the next one. Say something like: "in the recent years global sugery has become a field on its own with the goal of providing access to safe and affordable surgery for all"

Thank you! This has been added to the introduction.

Page 5, line 51… maybe start the sentence with "worldwide"
Thank you. Worldwide has been incorportated in the sentence.

Methods:
Page 7, line 22: dot missing
Corrected

Page 7, line 24. I would delete "major"
Corrected

Why have you decided 2013-2014? is there a specific reason? Were more recent data not available?
At the time of data collection, these were the most recent years. A follow up study is planned to determine variations over time.

Page 7, line 43. Does this mean you are considering circumcisions, inguinal and umbilical

For peer review only

hernias major surgeries? I would really try to find a different name for it cause "major" sounds wrong. It usually refers to an index case or to a long challenging surgery. I am not even sure you need to need an adjective at all to describe it. You can just talk about surgery in general.

We have made a distinction between major and minor surgical procedures as many minor procedures (including drainage of abscess, circumcision under local anaesthesia, suturing of wounds and similar) may be done in several different locations in the study hospitals and therefore it is not possible to capture all procedures carried out. The definition that we have used is "A major surgical procedure was defined as any procedure performed in a hospital operating theatre requiring regional or general anaesthesia, or profound sedation to control pain".

Page 8, line 29-34: you need to clarify which two hospitals you have excluded either here or in the results. Was it DH? Or RRH or NRH?

They were Regional Referral Hospitals. This has been updated in the methods section.

You need to state in methods that you will analyze data according to the region because you do so in the results.

Analysis by region has been added to the methods.

Should you not specify also that you collected information on the level of experience of the surgeon that performed the surgery? Eg: Consultants/Registrars/Medical officers/General surgeons vs paediatric surgeons. And provide the definition of each as you do for the anesthetic team.

This has been clarified in the methods section.

Page 7, line 33: you say "date, age, diagnosis, surgical procedure, surgeon, type of anaesthesia and level of anaesthetist" I think you can word it a bit better. First of all you never in the result mention the datse of surgery so you can remove that. Then you should say "age of the patient, diagnosis, type of surgical procedure performed, level of expertise of surgeon and anesthetist, type od anesthesia performed"

The date was used to determine number of procedures each year and it was also used for the imputation of missing data. Therefore, they should remain in the methods section.
We have corrected the phrasing of the sentence and also present volumes by year.

In the results you present your data according to the age categories and you have not mentioned in the methods section that you will be doing it.

We have updated the methods section to include analysis describing the basic characteristics of the patients.

You also need to specify that you are including orthopedic, ENT, cardiothoracic and urology conditions in your results as it is not given for granted in most countries. And again I believe it is crucially important you specify that in the results you will also look into who performed these surgeries. Was in the paeds surgeon or another specialist?

We have changed "paediatric surgery" to "surgery for children" throughout to indicate that we also include procedures that are normally not performed by paediatric surgeons. We have further stressed that we are also investigating who is performing the surgical procedures. Uganda has only 3 registered paediatric surgeons and there are international visiting paediatric surgeons from time to time. Therefore, the majority of surgical care for children is delivered by specialists in other disciplines and also by non-specialised physicians. This is

For peer review only

one of the things that we wanted to show with this study.

I do not think you need to specify in the methods what you mean for "met need" and "unmet need". You can expand this in the discussion but, as you do not talk about it in the results, I do not think it is a necessary paragraph in the methods section.
We have updated the methods according to this recommendation.

In general you present more data in the results than what you say you will do in the methods paragraph. I would suggest you have concordant information in the methods and in the results. Basically you need to use this section of the study to introduce exactly what you will talk about in the results section.
The aim of this study was to investigate volumes and types of surgery in children performed at the highest level hospitals in Uganda and characterize differences between hospital levels and geographic location. We have elaborated the methods section to better match the results presented.

Results:
Page 9, line 18. Why is the national hospital not included in the image?
All hospitals that contributed with data to the study are included in the figure legend.

Page 9, line 20: what does "factor of 34" mean?
This has been rephrased to "The most productive RRH performed 21 times more surgical procedures on children compared to the least productive RRH".

Page 9, line 26: rather than saying "young" please provide the mean/median age
This has been rephrased to "Of the children 43% (n=3422) were below five years-of-age. In the NRH, more than half of the children were below five years-old (Table 1). In all, 60.1% (n=4797) were boys."

Table 1:
the table legend is wrong. You are not only providing info regarding the gender and age distribution in this table. There is much more information and the table legend should reflect it. It is impossible to understand what the p-values are referring to. Maybe add * or ¶ close to the two numbers that are being compared.
The title has been rephrased to "Basic characteristics of patients and human resource performing surgery for children per hospital type***"***. The p-values have been clarified in the table.

Table 2:
Also in this case the table legend is wrong. You need to give a brief description of what the table is about.
The title has been changed to "Table 2. Indications for surgical procedures for children in the publica health care sector in Uganda 2013-2014"

Why have you decided the group pediatric surgical conditions in abdominal and inguinal? I do understand it is not easy to find these categories but could you not broadly say

For peer review only

"pediatric surgery conditions" and then list all of them without separating hernias and hydroceles from the abdominal conditions?
We have updated table 2 following this advice.

"imperforate anus" is not really an abdominal condition but should be placed in "colorectal". I believe that "other" with 975 cases is a bit too broad. Could you not somehow list some of the conditions you listed as "others"?
We have changed the title of this subgroup to also include colorectal conditions.

Why have you grouped together intestinal obstruction, intussusception and ruptured spleen?
Table 2 has been updated with new subheadings. They are grouped together as they represent abdominal conditions.

"neurological" should be replaced by "Neurosurgical".
Done

All the percentages refer to the total of each column which is not wrong but I think it would be interesting to know, per condition (hence per row) what percentage of cases were done in a DH, in a RRH and in a NRH.
Thank you for this suggestion. We agree that it is interesting to compare the different hospital types. The most interesting we thought, was to compare case mix and to do that, we needed to present the proportions per hospital type.

Table 3:
Table legend too short.
The title has been updated to "Table 3. Paediatric surgical procedures by hospital level in Uganda 2013-2014."

Please check spelling: posterior sagittal anorectoplast is missing a "y" and is not an abdominal surgery.
Corrected.

Ramstaedt's procedure is spelled worng and there is no comma separating it from the previous procedure.
Corrected.

The title of the fourth column is wrong (there is a "%" which should not be there)
Corrected.

Page 13, line 3: reading "Other procedures" makes the reader feel like you are referring to the row "other procedures" in Table 3. Change the sentence please.
Corrected.

Page 13, line 6: what is your definition of complex pediatric surgical conditions?
We agree that this can be difficult to define and have removed the sentence.

For peer review only

Page 13, line 15: "there was a mix of" reads very wrong. I would rather try and word it like the previous sentence and make it sound like "all neurosurgery cases were performed at NRH and some RRH" but you could also think of deleting this sentence as all of its content is already summarized in Table 3
This section has been updated. We have kept the latter part as it points out that neurosurgical procedures were performed only in some RRH (not very small volumes in all RRH).

Page 13, line 28: who belong to the "non-specialist physicians" group? Is it medical officers and interns? Why do you separate residents from them as they are also non-specialists?
We have added "other" to the sentence. The residents are not specialists, but they are focused in a particular field and represent the transition from a medical officer to a specialist. In addition, from a surgical training perspective it is interesting to know what and how much the residents operate.

Page 13, line 35: none of the procedures were performed by a pediatric surgeon? IS this right? If by "specialist in general surgery" you mean general paediatric surgeons than you should state it. It would also be very interesting to know how many general paediatric surgery procedures were performed by paediatric surgeons and how many by general adult surgeons if you know this information.
This study included several surgical conditions that are managed by different surgical specialities (paediatric surgery, ENT, plastic surgery, neurosurgery etc). Therefore, we have chosen to differentiate the human resource into specialists and non-specialists.

Page 13, line 54 to Page 14, line 4: Most of what you are saying is already summarized in the Table and the way you are saying is more in keeping with the discussion than with the results.
We have removed most part of this section.

Discussion:

Page 14, line 38-40: reference missing.
Reference has been added.

Page 14, line 52: how did you estimate this 5.6-8.8? I have tried to figure it out but it does not add up. Also I do not think you can really state this if you are not including all hospitals in Uganda as you clearly state at the beginning.
The estimate has been removed.

Page 16, line 42: I think you should also comment on trauma prevention.
Do you know how many of these kids were HIV positive or exposed when neonates? I believe also HIV prevention would play an important role in surgical prevention especially for acquired infective conditions such as perineal abscesses, HIV associated fistulae, anogenital warts… any opinion on that?
Prevention is very important, and we have added that to the discussion. We don't know the HIV prevalence of the children in the study.

English and general flow of the article is not very smooth and fluent.
The paper has been reviewed by a native English speaker for linguistic errors.

For peer review only

In summary, I believe it is an interesting study and that it has potential. I am very much aware that collecting data in LMIC countries is extremely challenging and you need to be complimented for the huge amount of work and information you put together. It will be a very good starting point for future retrospective and prospective studies. So, although this study does not add much data in terms of new conditions or new surgical approaches I still believe it created a good overview on the lack of healthcare workers in LMIC, especially of paediatric surgeons. Therefore, I believe it does deserve to be published but I do recommend some major revisions. Mainly it should read a bit more fluent, the material and methods section should introduce all the data presented in the results section and the results should be better explained and worded.

Good luck.

Reviewer: 2
Abstract
The results section needs to be more informative. Following details need to be provided:

- ***It's unlikely that the 4322 surgeries was the same for each year, hence the total surgeries for the 2 years need to be included***

We present the number for each year individually.

- ***The actual number and percentage that were emergent should be provided***

We agree that it would have been interesting but the exact number is not known. For some procedures, it is obvious, but for others it could have been both elective and emergent.

- ***Information regarding the different surgical specialties, non-physicians and the distribution and key aspects of patient characteristics should be summarized.***

The conclusion as provided is not based on the data presented in the results section of the abstract. For instance, there's no information on distribution, task sharing and actual numbers of paediatric surgeons.
We have updated the abstract. In particular we have clarified the abstract to further underscore the aspect of task sharing. 60% of the surgical procedures were performed by specialist physicians (thus 40% by non-specialist physicians), and anaesthesia was provided by specialist physician anaesthesiologists in 10% (thus 90% by non-specialist physicians and non-physician clinicians). This represents extensive task sharing/shifting.

What is already known
The focus should be on children, not the entire 5 billion that lack access. The number of children lacking access has already been estimated (Estimates of number of children and adolescents without access to surgical care. Mullapudi B, et al. Bull World Health Organ. 2019 Apr 1;97(4):254-258).
Thank you! This has been added to the introduction.

Introduction
Please see reference on estimated number of children lacking access and incorporate.
Done.

For peer review only

Methods

Why was the study restricted to 2013/2014? This restriction makes the data rather redundant as it's unlikely to provide a true reflection of current situation. To make this work helpful, I suggest the data is updated to 2019 or 2018. Moreover, the data predates 2015, since when there has been some progress in attention given to surgical care, following the work of the Lancet Commission on Global Surgery, World Bank's Disease Control Priorities 3 and World Health Assembly resolution 68:15.

At the time of data collection, these were the most recent years. We are aware of the increased awareness regarding global surgery since 2015. However, the Lancet commission and the WHA 68:15 has not resulted in much change of direction within the Ministry of Health and the health care system in Uganda. A follow up study is planned to determine variations over time.

How does met/unmet need come into the methodology? Given that this is a hospital-based study, these needs cannot be ascertained and not relevant to the methods of this report.

Unmet need has been removed from the methods section.

Information needs to be provided on the numbers of paediatric surgeons and Anaesthetists in each hospital and country as well as their regional distribution in the country.

This study was carried out in Uganda and includes conditions normally managed by different surgical disciplines (paediatric surgery, ENT, plastic surgery, neurosurgery etc). There are only three paediatric surgeons in Uganda and they are employed at the national referral hospital. We have also elaborated on the human resource per hospital type. This is specified in the methods section. We have chosen to present the data as number and proportion of the patients operated by a specialist in a surgical discipline.

The actual population figures for the <18 years population needs to be provided for the country as well as the regions/areas covered by the hospitals.

Additional information is needed on the structure and referral system in Uganda. For instance:

- ***What is the mandate for the national referral hospital, regional referral hospitals and district hospitals?***

Done

- ***What staffing with regards to children is expected to be deployed at each of these hospital levels?***

Done

- ***Including this information would help to better understand the data presented.***

Results

Since only 14 of the 38 district hospitals were included, the rates can only be calculated for the population covered by these hospitals/study and not the entire country.

The health care system is organised so that catchment populations overlap between the different levels of the hospitals. The National Referral Hospital has the entire population as its catchment population. The Regional referral hospitals were constructed for catchment populations of about 2 million and the general hospitals for around 500,000. This means that the NRH serves the entire population, the RRHs together serve the entire population, and the GH selected for this study serve part of the population. There is no part of the population that

For peer review only

we can remove from the calculations. However, we discuss the fact that we have not included all GHs in the study in the discussion.

Table 1
The p values need clarification.
Done.

There were 8644 surgeries, but the table shows 7886. If some patients had multiple procedures, the numbers should be provided in a footnote.
The figures represent patients. 7886 are the patients identified and after imputation this was 8238. We noticed an error in the previous analysis and therefore the figure after imputation has been altered.

Level of surgical training- among the specialists, it's not clear who is a paediatric surgeon. The residents also does not indicate who is a paediatric surgery resident. The same goes for anaesthesia.
We have rephrased the study title and the methods section. The study is about surgery for children, not only paediatric surgery. Several surgical disciplines routinely manage children. Therefore, we present number and proportion of procedures performed and anaesthesia administered by specialists, physicians of different training levels and non-physicians (anaesthesia only). There is only one peaediatric anaesthetist in Uganda and we have chosen not to present exactly what this one person does. From a health care systems perspective, it is not interesting and from an integrity perspective it would be questionable.

Table 3
It's not clear which procedures were elective or emergency surgery, even though some conclusion has been made on this.
The data often did not include information about emergency and elective care. Some conditions are by definition emergencies and that is why we concluded that emergency surgical care was common. To avoid confusion, we have removed this from the paper.

Discussion
As not all district hospitals were studied, the last 2 statements in the 2nd paragraph could be misleading.
This has ben rephrased.

As no real geographic provider density has been provided, the conclusion in the 3rd paragraph is not convincing.
Provider density is hard to investigate and varies over time in part due to how training is organised. For instance, one of the paediatric surgeons was employed in the national referral hospital but was away for training abroad at the time of the study. Surgical and anaesthesia residents train in the NRH, but are often employed at other hospitals that continue to pay their salaries. Surgery performed by visiting teams is also part of the data presented. In addition to this, it is very difficult to investigate the proportion of the time that surgeons and anaesthesiologists are available in their respective hospitals. Therefore, we have presented number and proportion of procedures performed by specialist physicians versus by health care providers with other levels of training and professions.

For peer review only

In the light of recent progress in global surgery and children's surgery, the discussion section needs to be significantly strengthened. It's important to discuss the role of national surgical, obstetrics and anaesthesia plans (NSOAPs) in scaling up surgical volumes and access to surgical care. Does Uganda already have an NSOAP or in process of developing one, and to what extent is/would children's surgical care be included?

It would help if the authors make more specific recommendations to increase paediatric surgical volumes and access to children's surgical care, beyond saying there should be investment. In this regard, they may find the optimal resources for children's surgery (OReCS) created by global initiative for children's surgery (GICS) helpful. The OReCS has provided a clear roadmap on scaling up children's surgical care in low and middle income countries (Optimal Resources for Children's Surgical Care: Executive Summary. Global Initiative for Children's Surgery. World J Surg. 2019 Apr;43(4):978-980) (Full details available from www.globalchildrenssurgery.org/optimal-resources/)

Uganda has a national plan for health care delivery. Surgical care is part of this plan. We have elaborated with more specific recommendations based on the study findings. We believe that it is wise to build on what already exists and therefore, task sharing and task shifting receives particular attention in the discussion. We have related this to the roadmap outlined in " *Optimal Resources for Children's Surgical Care: Executive Summary* ". We are aware of the increased attention of surgery in a global perspective and the extensive efforts invested into developing NSOAPs. It is still early to say which effect on surgical care for children (and adults) in terms of quantity and quality that the NSOAPs will have.

References
Some references are duplicated and some incomplete.
Corrected.

Overall, the data presented covers 2013/2014, predating the progress that has been made since 2015 in global surgery and children's surgery.
Much has already been published about access and capacity for children's surgery in Uganda. What the authors present here does not add anything new and may give the impression that no progress has been made, which may be misleading.

Thank you for your comments. While several advances on advocacy level and also policy level regarding surgery and paediatric surgery has been made, this has not (yet?) led to noticeable changes in Uganda. We politely disagree that a lot has been published about access and capacity for children's surgery in Uganda.

Reviewer: 3
The major limitation is that this study will underestimate the provision of paediatric surgical services in Uganda given the nongovernmental sector covers a large proportion of surgical services. This should be made clearer in the abstract, discussion and article summary.
This study was designed to investigate surgical provision for children in the public health care sector. This has been clarified in the methods section and is mentioned again in the discussion. We have elaborated further on the capacity in the study hospitals and relate it to the estimated need (based on the previous population-based paper by Butler et al) for surgery for children. We believe that the remaining general hospitals and the private health care

For peer review only

sector are unlikely to be able to compensate for the low rates within the public health care system.

The authors should expand on comparison of this study (case mix, rate of major surgical procedures etc.) with data from previous Ugandan studies e.g. reference 4. in the discussion.

Thank you, we have elaborated on the comparison. The main difference is that the differentiation between major and minor surgery was not the same. In the present study, we have not included wounds and abscesses, for instance. The reason for this is that these conditions represent a spectrum if severity, of which most probably require small surgical interventions but the data is not granular enough to distinguish large wounds and abscesses from small ones. In addition, many of these are be treated in other locations in a hospital apart from the operating room and may not always appear in the operating room record.

The authors suggest task-shifting as a solution to increasing the provision of surgery, but should address it's limitations in the discussion more fully. E.g. importance of perioperative care on outcomes should be discussed, best outcomes are in high volume centres, type of postoperative care available (HCU, NICU, PICU) which will limit types of surgery and patients, limited equipment, infrastructure and physical resources in rural areas etc.

This study did not include a survey of the facilities, availability of water, electricity and other essentials for surgical care. However, we did investigate the human resource providing the surgical services and document widely implemented task sharing and task shifting where non-specialists are performing major surgery on children and non-physician clinicians administer most of the anaesthesia for surgery in children. We do not suggest task sharing and task shifting as a novel solution. It is already part of the solution and is implemented in routine surgical care since many years. In this study, we did not investigate outcomes after surgery. It would be very valuable to investigate the safety and effectiveness of the task sharing and task shifting that is practiced.

Were cases done under local anaesthesia included when calculating met need? (See definition of major surgery). Were no cases performed under sedation?

We have removed the calculation for met need and instead we present surgical rate. The definition for major surgery was … "that usually require general or regional anaesthesia or profound sedation to control pain". For example, all hernia repairs were included even though some may have been performed under local anaesthesia (+/- sedation).

The national referral hospital is effectively providing a specialist service to children from the whole of Uganda. When calculating the rates per region was this accounted for? The paediatric population number used to calculate rates should be included.

Thank you for this comment. We have updated the calculations and allocated the surgery performed at the national referral hospital to the four regions of Uganda. This distribution has been done based on a previous publication from the paediatric surgery registry at Mulago National Referral Hospital.

Table 1. Suggest adding a row to summarise numbers & include relative percentage of cases between different levels of hospital

Thank you for this suggestion. We agree that it is interesting to compare the different hospital

For peer review only

types. The most interesting we thought, was to compare case mix and to do that, we needed to present the proportions per hospital type.

Reviewer: 4

1. Abstract: The conclusions are not informed by the study results.

The abstract has been updated accordingly.

2. Study Methodology: The methodology is not clear. Sample size determination and sampling of the hospitals that were included into the study were improper, hence the sample was not representative enough to enable inference and generalization of the study results.

We included the national referral hospital (there is only one for somatic conditions), all regional referral hospitals and an equal number of district hospitals (one district hospital per regional referral hospital), representing the highest-level hospitals in the public health care sector in Uganda. This has been clarified in the methods section. There was no sample size calculation as all major procedures carried out during the study period were included. The internal validity is high, for these reasons.

3. How did you choose only 14 district hospitals out of 113?

In Uganda, there were 38 public district hospitals at the time of the study. The rest are private facilities of different levels. In Uganda, also large private hospitals will be categorised as district hospitals by the Ministry of Health. We chose one hospital per regional referral hospital based on productivity and convenience. In several cases, there was only one district hospital available in the catchment area of a particular RRH.

4. Where is the data of the children whose "needs were unmet"?

We have updated the calculation and present it in Table 1.

For peer review only

Surgical procedures for children in the public health care sector – a nationwide, facility-based study in Uganda

Journal:	*BMJ Open*
Manuscript ID	bmjopen-2020-048540.R1
Article Type:	Original research
Date Submitted by the Author:	31-May-2021
Complete List of Authors:	Ajiko, Mary Margaret; Soroti Regional Referral Hospital, Surgery<br>kressner, Julia; 1. Department of molecular medicine and Surgery, Karolinska Institute,<br>Matovu, Alphonsus; Mubende Regional Referral Hospital, Surgery; Karolinska Institute, Molecular Medicine and Surgery<br>Nordin, P.; Umeå University, Department of Surgical and Perioperative Sciences; Östersunds sjukhus, department of surgery<br>Wladis, Andreas; Linköping university, Department of Clinical and Experimental Medicine<br>Löfgren, Jenny; Karolinska Institute, Molecular Medicine and Surgery; Karolinska University Hospital, Reconstructive Plastic Surgery
**Primary Subject Heading**:	Surgery
Secondary Subject Heading:	Global health
Keywords:	SURGERY, PUBLIC HEALTH, PAEDIATRIC SURGERY

SCHOLARONE™ Manuscripts

I, the Submitting Author has the right to grant and does grant on behalf of all authors of the Work (as defined in the below author licence), an exclusive licence and/or a non-exclusive licence for contributions from authors who are: i) UK Crown employees; ii) where BMJ has agreed a CC-BY licence shall apply, and/or iii) in accordance with the terms applicable for US Federal Government officers or employees acting as part of their official duties; on a worldwide, perpetual, irrevocable, royalty-free basis to BMJ Publishing Group Ltd ("BMJ") its licensees and where the relevant Journal is co-owned by BMJ to the co-owners of the Journal, to publish the Work in this journal and any other BMJ products and to exploit all rights, as set out in our licence.

The Submitting Author accepts and understands that any supply made under these terms is made by BMJ to the Submitting Author unless you are acting as an employee on behalf of your employer or a postgraduate student of an affiliated institution which is paying any applicable article publishing charge ("APC") for Open Access articles. Where the Submitting Author wishes to make the Work available on an Open Access basis (and intends to pay the relevant APC), the terms of reuse of such Open Access shall be governed by a Creative Commons licence – details of these licences and which Creative Commons licence will apply to this Work are set out in our licence referred to above.

Other than as permitted in any relevant BMJ Author's Self Archiving Policies, I confirm this Work has not been accepted for publication elsewhere, is not being considered for publication elsewhere and does not duplicate material already published. I confirm all authors consent to publication of this Work and authorise the granting of this licence.

For peer review only

Surgical procedures for children in the public health care sector – a nationwide, facility-based study in Uganda

Mary Margaret Ajiko (MD)[1,2], Julia Kressner (MD)[1], Alphonsus Matovu (MD)[3], Pär Nordin (PhD)[4], Andreas Wladis (PhD)[5], Jenny Löfgren (PhD)[1]

1. Department of Molecular Medicine and Surgery, Karolinska Institute, Sweden
2. Soroti Regional Referral Hospital, Uganda
3. Mubende Regional Referral Hospital, Uganda
4. Department of Surgery and Perioperative Sciences, Umeå University, Sweden
5. Department of Clinical and Experimental Medicine, Linköping University, Sweden

Corresponding author

Dr Jenny Löfgren,

1. Department of Molecular Medicine and Surgery, Karolinska Institutet.

Karolinska University Hospital L1:00. 17176 Stockholm, Sweden.

2. Karolinska University Hospital, Reconstructive Plastic Surgery

Stockholm, SE 171 76

e-mail: jenny.lofgren@ki.se

Word Count: 3144

For peer review only - http://bmjopen.bmj.com/site/about/guidelines.xhtml

Abstract

Objective

This study investigated the surgical services for children at the highest levels of the public healthcare sector in Uganda. The aim was to determine volumes and types of procedure performed, the patients and the human resource involved.

Design

The study was a facility-based, record review.

Setting

The study was carried out at the National Referral Hospital, all 14 regional referral hospitals and 14 general hospitals in Uganda, representing the highest levels of hospital in the public healthcare sector.

Participants

The subjects were children <18 years who underwent major surgery in the study hospitals during 2013 and 2014.

Results

The study hospitals contribute with an average annual rate of paediatric surgery at 22.0 per 100,000 paediatric population. This is a fraction of the estimated need. Most of the procedures were performed for congenital anomalies (n=3111, 39.4%), inflammation and infection (n=2264, 28.7%), and trauma (n=1210, 15.3%). Specialist surgeons performed

For peer review only - http://bmjopen.bmj.com/site/about/guidelines.xhtml

60.3% (n=4758) of the procedures and anaesthesia was administered by specialist physician anaesthetists in 11.6% (n=917) of the cases.

Conclusions

A variety of paediatric surgical procedures are performed in a relatively decentralised system throughout Uganda. Task shifting and task sharing of surgery and anaesthesia are widespread: a large proportion of surgical procedures were carried out by non-specialist physicians, with anaesthesia mostly delivered by non-physician anaesthetists. Reinforcing the capacity and promoting the expansion of the health facilities studied, in particular the general hospitals and regional referral hospitals, could help reduce the immense unmet need for surgical services for children in Uganda.

For peer review only

For peer review only - http://bmjopen.bmj.com/site/about/guidelines.xhtml

Article summary

Strengths and limitations of this study

- This study included information about surgical procedures carried out in children in 29 hospitals (primary, secondary and tertiary institutions) within the public health care sector all over Uganda.
- The main limitation of the study is the retrospective study design and that the level of detail was limited to that of the logbooks reviewed.
- The data collected is now (2021) 7-8 years old, preceding recent global surgery efforts. Changes over time can be evaluated in relation to the findings in the present study.

For peer review only - http://bmjopen.bmj.com/site/about/guidelines.xhtml

Introduction

In recent years, global surgery has become a field of its own with the goal of providing access to safe and affordable surgery for all. In 2017, 1.7 billion children and adolescents lacked access to surgical care (1). Many childhood surgical conditions can be treated successfully with surgery, while failure to do so may lead to lifelong complications, disability, or even death (2)(3). The provision of surgical services for children is highly cost-effective (4).

Children requiring surgical intervention make up a significant proportion of child admissions. Information available suggests that 6-12% of all paediatric admissions in sub-Saharan Africa are surgical, although the proportion may be higher in some urban areas (5). In a regional referral hospital in Uganda, a third of the patients admitted to a surgical department during a three-year period were below 18 years of age (6). This however is not enough to meet the need for surgical services for children. In a cross-sectional survey performed in Uganda, Sierra Leone, Rwanda and Nepal, 19% of children had a surgical condition and 62% of these children had at least one unmet surgical need (3). A study in Uganda found that 7.4% of children were currently suffering from an untreated surgical condition (7).

Uganda is a sub-Saharan, low-income country with a population of over 20 million children (8). With a birth rate of 5.0 children per women, the paediatric population is rapidly increasing (9). Although 80% of the Ugandan population live in rural areas, 95% of surgeons practice in an urban environment(10). The extent to which the healthcare system can provide surgical services for children at national level is not known. The objective of the study was to investigate volumes and types of paediatric surgery performed at the highest-level hospitals

For peer review only

in Uganda, to relate that to the need for surgery in the paediatric population and to describe the human resource delivering the services.

Method

Study design

This was a retrospective review of relevant hospital records.

Study location, study population and study size

Some 29 hospitals geographically distributed throughout Uganda were included. These hospitals represent the highest level of public health care and include the National Referral Hospital (Mulago), all 14 regional referral hospitals (RRH) and an additional 14 general hospitals (GH) (Figure 1). Data from two RRH were incomplete and they were therefore excluded from the analysis.

The public healthcare system in Uganda is divided into seven levels: from village health teams (level I) to health centres (levels II-IV) and hospitals (levels V-VII). The hospitals are divided into general (district) hospitals (GH, level V), regional referral hospitals (RRH, level VI) and the National Referral Hospital (NRH, level VII). All private hospitals, are classified as level V hospitals. Uganda has 113 general hospitals, 38 of which are public-service hospitals. The rest are private and private non-profit hospitals (11). Each RRH has at least one general hospital in its catchment area. Due to scarcity of suitable public general hospitals in the northern and eastern regions, two NGO general hospitals were included in the study.

For peer review only - http://bmjopen.bmj.com/site/about/guidelines.xhtml

The catchment populations between the GHs, RRHs and NRH overlap as they are meant to provide services of increasing complexity. The general hospitals and the regional referral hospitals were designed for a catchment population of 500,000 people and two million people respectively, but these hospitals cover much larger populations now due to rapid population growth. GHs have specialists in internal medicine, gynaecology and obstetrics and in paediatrics, but not in surgery. Non-specialist medical doctors perform surgical procedures. Each RRH has a mandate to receive referred patients from the regions they serve, but self-referral from other regions is also permitted. The RRHs employ general surgeons. The National Referral Hospital covers the entire population for highly specialised services. It is also a teaching facility of Makerere College of Health Sciences. The three paediatric surgeons and the paediatric anaesthetist in Uganda are employed at this institution.

Since 2001, all public healthcare facilities have been officially free of charge (12), which is why the public healthcare sector was selected for this survey. To describe the surgical activity for children here, the NRH and all the 14 RRHs were included. In addition, one general hospital per RRH was selected. It was hypothesized that the surgical activity for children in the GHs would be low, as no surgeons are employed in these hospitals. This selection of hospitals allowed comparisons between the three highest levels in the public health care sector.

Logbooks from operating theatres for the years 2013 and 2014 at the hospitals studied were photographed and the information extracted was entered into excel spreadsheets. Data on all major surgical procedures performed on children were included. The logbooks provided, for each hospital, information about children that had undergone a surgical procedure. They included date of procedure, age of the patient, diagnosis, type of surgical procedure

For peer review only - http://bmjopen.bmj.com/site/about/guidelines.xhtml

performed, names of surgeon and anaesthetist, and type of anaesthesia used. Each surgeon's and anaesthetist's training level was thereafter clarified with each hospital. In this study, a child is defined as "a human being below the age of 18 years", according to the United Nations Convention on Children's Rights. This is also in keeping with the Ugandan Constitution (13). In 2014, Uganda children 0-17 represented 55.1% of the population, or 19.1 million children (14).

A major surgical procedure was defined as "any procedure conducted in the operating theatre involving the incision, excision, manipulation or suturing of tissue which usually requires regional or general anaesthesia or profound sedation to control pain"(15). An anaesthetic assistant/officer was a healthcare provider who had undertaken a 2-3 year course in anaesthesia at a recognised institution, and had been certified by the Allied Health Professionals Council of Uganda (16). A senior house officer (SHO) was a resident in surgery or anaesthesia. An intern had completed medical school and formally practices under the supervision of a senior colleague. A specialist physician is a doctor who has completed advanced education and training in a specific field of medicine.

Data analysis

Data were analysed by region and by hospital type using standard descriptive methods. Data are presented to describe the basic characteristics of the patients, the procedures and the human resource providing the anaesthesia and surgical services. Analyses used for group comparisons were Pearson's *t* test for continuous data and the Chi square test for proportions and identification of associations between demographic characteristics and indications for surgery. We also categorised gender and age distribution with the number of procedures performed on children for each hospital level. Missing data on the number of procedures

For peer review only - http://bmjopen.bmj.com/site/about/guidelines.xhtml

performed was handled by multiple imputation. The surgical volumes at the NRH were distributed to the entire population with the relative proportion of 74% to the central region, 12% to the western region,13% to the eastern region and 1% to the northern region. This distribution was based on previous research on geographical distribution of paediatric surgical patients presenting to the NRH (17). The 2014 national population census for Uganda were used to calculate the rate of surgical procedures for children per 100,000 paediatric population for 2014. The paediatric population of Uganda for 2013 was calculated based on the 2014 national census and a population growth of 3.3% per year (18).

In order to relate the hospital surgical productivity to the need for surgery, a population-based study on paediatric surgical need carried out in Uganda in 2014 was used (7).

For identification of common diagnoses and procedures, only complete original data were used. Two regional referral hospitals were excluded from the analyses; one had no data for the entire study period and the other omitted patient age in the majority of cases, making it impossible to extract all paediatric cases.

Ethical considerations

Ethical approval was granted by the Makerere University School of Public Health (HDREC 076) and the Uganda National Council of Science and Technology (HS1892). The Medical Superintendent of each hospital approved collection of data from the theatre log books. The retrospective nature of the study meant that the treatment delivered to the patients was not affected. To protect their integrity, patients' names were not included.

For peer review only - http://bmjopen.bmj.com/site/about/guidelines.xhtml

For peer review only

Role of the funding source

The funder of the study had no role in the study design, data collection, data analysis, data interpretation, writing of the article or the decision to submit for publication. All authors had full access to all the data in the study and were responsible for the decision to submit for publication.

Patient and Public involvement

There was no involvement of patients or the public.

Results

A total of 7886 children who had undergone a major surgical procedure were identified in the theatre records of the 27 hospitals during the study period. After imputation for missing data, this number was 8238 of which 4680 were operated in 2013 and 3558 in 2014. This represented an average rate of 22.0 major surgical procedures per 100,000 paediatric population of Uganda in 2013-2014. The rate varied between the four regions in Uganda: from 12.0 per 100,000 children in the northern region to 27.7 per 100,000 children in the central region (Figure 1). There was considerable variation in surgical volumes within the RRH and GH groups and the most productive RRH performed 21 times more surgical procedures on children than the least productive.

A population-based study in Uganda previously investigated the prevalence of surgical conditions and the need for surgical consultation and surgical intervention among children in Uganda (7). In relation to the findings of this previous study, the study hospitals in the

For peer review only - http://bmjopen.bmj.com/site/about/guidelines.xhtml

present study contributed with surgical volumes corresponding to 4.9-7.6% of the need for major surgical procedures in children (Table 1).

The basic characteristics of the patients and the surgical and anaesthesia providers are shown in Table 2. Of the children, 43.4% (n=3422) were below five years of age, and 60.1% (n=4797) were boys. In Appendix 1, the age distribution by hospital level is visualised. Most of the procedures (n=4758, 60.3%) were performed by specialists in general surgery or specialists in other surgical specialities (paediatric surgery, gynaecology, orthopaedic surgery, cardiothoracic surgery, plastic surgery, neurosurgery, ear nose and throat surgery and urology). Overall, surgical residents performed 14.5% (n=1145) of the procedures and other non-specialist physicians performed 20.5% (n= 1616). Interns carried out 3.0% (n=237). Anaesthesia was rarely administered by a physician anaesthetist (n=917, 11.6%) or physician anaesthetic resident (n=818, 10.4%). The majority of the patients were anaesthetised by an anaesthetic assistant or anaesthetic officer (n=5469, 69.4%).

The surgical procedures were performed by 275 medical doctors with different levels of qualification: 103 specialist surgeons in different disciplines of whom three were paediatric surgeons, 57 resident surgeons in different surgical disciplines and 115 medical officers without specific training in surgery. Of the specialists, 40 performed surgery at the NRH and 61 at the RRH. Only two specialist surgeons performed surgical procedures at a GH. Anaesthesia was administered by 18 physician anaesthetists and 99 anaesthetic officers. Of the physician anaesthetists, three worked at a RRH and 15 at the NRH. No physician anaesthetist worked at a GH.

For peer review only - http://bmjopen.bmj.com/site/about/guidelines.xhtml

Indications for the surgical procedures are presented in Table 2. The most common were related to congenital anomalies (n=3111, 39.4%) infectious and inflammatory conditions (n=2264, 28.7%) and trauma (n=1210, 15.3%). Table 3 shows the surgical procedures that were carried out. Abdominal and colorectal surgical procedures were most common, followed by orthopaedic and ear, nose and throat (ENT) procedures. The types of procedure varied, and in the GH hernia surgery was the most common. Surgery for congenital heart conditions (n=128, 1.6%) was only performed at the NRH where there is a heart institute run by cardiothoracic surgeons. Neurosurgeons performed all neurosurgical procedures including ventriculoperitoneal shunts, spina bifida repair, brain abscess drainage, craniotomy and meningocele repair. Such procedures were carried out at the NRH (Mulago Hospital) and some RRHs.

Discussion

The study hospitals in the present study contributed with an average annual surgical rate of 22.0 major surgical procedures per 100,000 paediatric population in Uganda in 2013 and 2014. The patients were often below five years and the surgical procedures were mainly carried out for congenital anomalies, inflammatory and infectious conditions and trauma. Task sharing and task shifting were widely practised as non-specialists frequently performed surgical procedures and as non-physician clinicians administered anaesthesia in most of the cases.

A considerable variation in the number and rate of major procedures performed at the different hospitals, and within the four regions of the country, was seen. The northern region

For peer review only - http://bmjopen.bmj.com/site/about/guidelines.xhtml

For peer review only

was the most underserved, where hospitals are few and the distance to the national referral hospital is greatest. This is similar to the results of a previous study in Uganda, which found a positive correlation between burden of paediatric surgical disease and distance to the nearest healthcare unit offering surgery for children (19). The majority of the Ugandan population live in a rural setting with very limited financial resources (6)(20). Transport costs are likely to pose a barrier to surgical care for children there. This is confirmed by previous research in low- and middle-income countries showing that cost and travelling distance are barriers to surgical care access (21). Decentralised services are therefore needed.

The Global Initiative for Children's surgery developed the Optimal Resources for Children's Surgical Care, published in 2019. This document stratifies by level of hospital what services and human resource should be available at each level. The recommendations are in line with what is demonstrated in the present study. On GH level, intermediate surgical services, including hernia repair and acute abdominal and traumatic conditions, should be performed (22). In the general hospitals in the present study, hernia surgery represented the vast majority of procedures performed, followed by other abdominal and orthopaedic procedures. These procedures were carried out on all three levels but in addition, the RRHs and NRH performed procedures of increasing complexity including surgical subspecialities.

For essential surgical care to be accessible for all children in Uganda, service delivery in both GHs and RRHs should be further promoted. It is encouraging to see that surgery for children was delivered in these hospitals even if at rates that represent only a fraction of the need. The more productive hospitals in each group could serve as models for the less productive ones. A first goal would be for all hospitals on the same level to deliver the same services and similar volumes of surgery. The present study did not investigate the outcomes after surgery,

For peer review only - http://bmjopen.bmj.com/site/about/guidelines.xhtml

and this should be evaluated in future research. Volume and quality represent two sides of the same coin and capacity-building must take both into consideration.

Significantly more boys than girls were recorded in this study. Several of the most common diagnoses requiring surgery were gender-specific, or over-represented, in males. For example, inguinal hernia, which was the most common indication for surgery in the GHs, is more common in boys than in girls (23). Female-specific diagnoses such as gynaecological conditions were rare. This study could not determine whether boys have better access to surgery than girls, but future research into gender as a barrier to surgical care in children could investigate this further.

Task-sharing and task-shifting of surgical duties and anaesthesia delivery were routinely practiced in the study hospitals. Task sharing with medical doctors without specific training in surgery is common in many countries but the safety and effectiveness as well as the training available for this important group of surgical providers is not well understood (24). Ways in which the highest possible quality of paediatric surgical services can be achieved and maintained must be the subject of future research. Standardization of the surgical training, and support and monitoring of non-surgeons for the commonest surgical conditions should be prioritized. The anaesthetic officers in Uganda have formal training and their duties are probably better regulated than those of non-specialist medical doctors. Nonetheless, regular monitoring of the surgical and anaesthetic work forces and the institutions in which they work is an essential component of the provision of high-quality and cost-effective surgical services for children. An additional important role of the health care system is to promote prevention: many surgical conditions can be prevented. Antenatal care and

For peer review only - http://bmjopen.bmj.com/site/about/guidelines.xhtml

vaccinations are carried out in the study hospitals and health information and child safety issues could be discussed with parents during such visits.

Surgery has an important place in several of the health targets in the UN's Sustainable Development Goal 3 (25). Uganda has a national plan for service standards, and service delivery standards for the health sector include a package for surgical, obstetrics, anaesthesia plans in scaling up surgical volumes and access to surgical care. However, a defined paediatric surgical package is not included (26). To reduce the death toll and disability caused by surgical conditions in the paediatric population, the healthcare system must be improved in order to deliver safe surgery and prevention strategies. Advancing and promoting the service delivery already in place could be part of the solution to reach the goal of surgical services of quality for all children with surgical conditions.

Strengths and Limitations

This study set out to investigate the situation for surgery for children in the public healthcare system in Uganda and to describe patients and their surgical providers at the three highest levels of hospitals. The study has several limitations. Due to limited resources, not all hospitals in the country could be visited and therefore overall volumes and surgical rates could not be calculated. The hospitals studied were the healthcare facilities first in line for the implementation of new policies of intervention, where Ministry of Health policy as well as increased funding would have a direct effect. Our choice of hospitals was thus relevant regarding current capacity and surgical volume, providing baseline information that is essential in the future evaluation of the effects of investment and interventions made to enhance and expand paediatric surgical services.

For peer review only - http://bmjopen.bmj.com/site/about/guidelines.xhtml

Given the retrospective collection of data in this study, the level of detail is limited to that of the logbooks reviewed. Information on outcome after paediatric surgery in relation to level of hospital and level of training of the surgical and anaesthesia providers would be very valuable. Where data on volumes of surgery performed during the study period were missing, in the selected hospitals, these were imputed as the best option. However, the data sufficed to provide important information about the state of surgery for children in Uganda.

The present data were collected five years ago and since then no significant investments have been made for improving surgical services for children on national level. There is still (2021) only one paediatric anaesthetist, three registered paediatric surgeons, and two hospitals with dedicated paediatric operating theatres. The findings should thus still apply today.

Acknowledgements

The authors acknowledge the invaluable assistance of the hospital directors and staff which made the study possible. The authors would like to thank Anna Peterson, William Yu and Andreas Vikholm for their important contribution in data collection and management. We also thank Dr Matteo Bottai for statistical assistance.

Funding

This work was supported through a grant from the Department of Public Health and Clinical Medicine, Unit of Research, Education, and Development, Östersund, Umeå University and by the Swedish Research Council.

Data sharing

For peer review only - http://bmjopen.bmj.com/site/about/guidelines.xhtml

Data may be shared upon request to the corresponding author.

Conflict of competing interests

There are no personal conflicts of interest or financial conflicts of interest among the authors.

Authors' Contributions

Study conception and design:
Jenny Löfgren, Andreas Wladis, Ajiko Mary Margaret, Pär Nordin.

Data acquisition:
Ajiko Mary Margaret, Jenny Löfgren, Julia Kressner, Alphonsus Matovu

Analysis and data interpretation:
Ajiko Mary Margaret, Jenny Löfgren, Julia Kressner, Andreas Wladis, Pär Nordin, Alphonsus Matovu

Drafting of the manuscript:
Ajiko Mary Margaret

Critical revision,
Ajiko Mary Margaret, Jenny Löfgren, Andreas Wladis, Par Nordin, Alphonsus matovu, Julia Kressner.

References

1. Mullapudi B, Grabski D, Ameh E, Ozgediz D, Thangarajah H, Kling K, et al. Estimates of number of children and adolescents without access to surgical care. Bull World Health Organ. 2019 Apr 1;97(4):254–8.

2. Walker IA, Obua AD, Mouton F, Ttendo S, Wilson IH. Paediatric surgery and anaesthesia in south-western Uganda: a cross-sectional survey. Bull World Health Organ. 2010 Dec 1;88(12):897–906.

3. Bickler SW, Rode H. Surgical services for children in developing countries. Bull World Health Organ. 2002;80:829–35.

4. Saxton AT, Poenaru D, Ozgediz D, Ameh EA, Farmer D, Smith ER, et al. Economic Analysis of Children's Surgical Care in Low- and Middle-Income Countries: A Systematic Review and Analysis. PloS One. 2016;11(10):e0165480.

For peer review only - http://bmjopen.bmj.com/site/about/guidelines.xhtml

5. Linden AF, Sekidde FS, Galukande M, Knowlton LM, Chackungal S, McQueen KAK. Challenges of surgery in developing countries: a survey of surgical and anesthesia capacity in Uganda's public hospitals. World J Surg. 2012 May;36(5):1056–65.

6. Tumusiime G, Was A, Preston MA, Riesel JN, Ttendo SS, Firth PG. The Quality and Utility of Surgical and Anesthetic Data at a Ugandan Regional Referral Hospital. World J Surg. 2017 Feb;41(2):370–9.

7. Butler EK, Tran TM, Fuller AT, Brammell A, Vissoci JR, de Andrade L, et al. Quantifying the pediatric surgical need in Uganda: results of a nationwide cross-sectional, household survey. Pediatr Surg Int. 2016;32(11):1075–85.

8. Population ages 0-14, total - Uganda | Data [Internet]. [cited 2020 Dec 22]. Available from: https://data.worldbank.org/indicator/SP.POP.0014.TO?locations=UG

9. Fertility rate, total (births per woman) - Uganda | Data [Internet]. [cited 2020 Dec 22]. Available from: https://data.worldbank.org/indicator/SP.DYN.TFRT.IN?locations=UG

10. Andersson O, Radell P, Ringo V, Mulungu M, Baker T. Quality of pediatric anesthesia: A cross-sectional study of a university hospital in a low-income country. PloS One. 2018;13(4):e0194622.

11. Kisa P, Grabski DF, Ozgediz D, Ajiko M, Aspide R, Baird R, et al. Unifying children's surgery and anesthesia stakeholders across institutions and clinical disciplines: challenges and solutions from Uganda. World J Surg. 2019;1–15.

12. Uganda Ministry of Health. Health Sector Strategic Plan 2000/01 - 2004/05 [Internet]. Available from: https://afri-can.org/wp-content/uploads/2016/04/HSSP-2000-01.pdf

13. Children Act. In: Ugandan Constitution. 1997.

14. Uganda Bureau of Statistics. National Population and Housing Census 2014 - Main Report [Internet]. [cited 2018 Jun 9]. Available from: http://www.ubos.org/onlinefiles/uploads/ubos/NPHC/NPHC%202014%20FINAL%20RESULTS%20REPORT.pdf

15. World Health Organization. Safe surgery saves lives. In: Second global patient safety challenge. 2008. p. 4.

16. Allied Health Professionals Council. The Allied Health Professionals Act - Chapter 268. In: The Allied Health Professionals Act.

17. Ullrich SJ, Kakembo N, Grabski DF, Cheung M, Kisa P, Nabukenya M, et al. Burden and Outcomes of Neonatal Surgery in Uganda: Results of a Five-Year Prospective Study. J Surg Res. 2020;246:93–9.

18. Population growth (annual %) - Uganda | Data [Internet]. [cited 2020 Dec 22]. Available from: https://data.worldbank.org/indicator/SP.POP.GROW?locations=UG

For peer review only

For peer review only - http://bmjopen.bmj.com/site/about/guidelines.xhtml

19. Smith ER, Vissoci JRN, Rocha TAH, Tran TM, Fuller AT, Butler EK, et al. Geospatial analysis of unmet pediatric surgical need in Uganda. J Pediatr Surg. 2017;52(10):1691–8.

20. Poverty headcount ratio at USD3.10 a day (2011 PPP) (% of population) [Internet]. The World Bank; 2012. Available from: data.worldbank.org/indicator/SI.POV.NAHC?locations=UG

21. Grimes CE, Bowman KG, Dodgion CM, Lavy CBD. Systematic Review of Barriers to Surgical Care in Low-Income and Middle-Income Countries. World J Surg. 2011 May;35(5):941–50.

22. Optimal Resources for Children's surgery [Internet]. Global Initiative for Children's Surgery. [cited 2020 Dec 6]. Available from: https://www.globalchildrenssurgery.org/optimal-resources/

23. Burcharth J, Pedersen M, Bisgaard T, Pedersen C, Rosenberg J. Nationwide Prevalence of Groin Hernia Repair. PLOS ONE. 2013 Jan 14;8(1):e54367.

24. Falk R, Taylor R, Kornelsen J, Virk R. Surgical Task-Sharing to Non-specialist Physicians in Low-Resource Settings Globally: A Systematic Review of the Literature. World J Surg. 2020 May;44(5):1368–86.

25. Home — SDG Indicators [Internet]. [cited 2020 Aug 19]. Available from: https://unstats.un.org/sdgs/#

26. HEALTH SECTOR DEVELOPMENT PLAN 2015/16 - 2019/20. :110.

For peer review only

Table 1. Surgical need and contribution towards the met need by the study hospitals

	Need for surgical consultation	Need for surgical intervention	Children operated in study hospitals	Interpretation
**Proportion of children**	7.4% *	7.6% - 11.8% of children with need for surgical consultation *	4.9 - 7.6 % of estimated need for a major surgical procedure	The 8238 children operated correspond to 4.9-7.6% of the estimated need for a major surgical procedure in the paediatric population of Uganda in 2014
**Number of children**	1,413,400 * (7.4% of 19.1 million paediatric population in 2014)	107,418 – 166,781 *	8238	Another 99,000 - 159,000 surgical procedures are needed to meet the need for major surgical interventions in children in Uganda
**Rate, n per 100,000 paediatric population**	7400 *	562 – 873 *	22.0	The surgical rate contributed by the study hospitals is a fraction of the estimated need.

*** Reference 7. Butler et al**

For peer review only

Table 2. Basic characteristics of patients and human resource performing surgery for children per hospital type

	General hospital, n=1276	Regional Referral Hospital, n=4475	National Referral Hospital, n=2135	Total n=7886	p-value
**Sex, n (%)**					
Male	945 (74.1)	2516 (56.2)	1336 (62.6)	4797 (60.8)	<0.001*
Female	324 (25.4)	1620 (36.2)	756 (35.4)	2700 (34.2)	
Not indicated	7 (0.5)	339 (7.6)	43 (2)	389 (5.0)	
**Age distribution (years)**					
Mean (SD)	7.4 (5.5)	7.1 (5.5)	5.8 (5.7)	5.3 (4.7)	<0.001*
<5	488 (38.2)	1790 (40.0)	1144 (53.6)	3422 (43.4)	
>5	777 (60.9)	2677 (59.8)	980 (45.9)	4434 (56.6)	
Not indicated	11 (0.9)	8 (0.2)	11 (0.5)	30 (0.4)	
**Level of training of surgical provider**					
Specialist surgeon (including 3 paediatric surgeons)	324 (25.4)	3063 (68.4)	1371 (64.2)	4758 (60.2)	<0.001*
Resident	0	437 (9.8)	708 (33.2)	1145 (14.5)	
Medical officer	904 (70.8)	700 (15.6)	12 (0.6)	1616 (20.5)	
Intern	0	232 (5.2)	5 (0.2)	237 (3.0)	
Not indicated	48 (3.8)	43 (1.0)	39 (1.8)	130 (1.6)	
**Level of training of Anaesthesia provider**					
Physician Anaesthetists, (one paediatric anaesthesiologist)	0	807 (18.0)	110 (12.0)	917 (11.6)	<0.001*
Residents in anaesthesia,	0	163 (3.6)	655 (30.7)	818 (10.4)	
Self/by surgeon local anaesthesia	47 (3.7)	162 (3.6)	10 (0.5)	219 (2.8)	
Anaesthetic assistant or Officer	1190 (93.3)	3132 (70)	1147(53.7)	5469 (69.4)	
Not indicated	39 (3.1)	211 (4.7)	213 (10)	463 (5.9)	
**Type of Anaesthesia**					
General anaesthesia	1097 (86.0)	3811 (85.2)	1913 (89.6)	6821 (86.5)	<0.001*
Spinal anaesthesia	66 (5.2)	186 (4.2)	82 (3.8)	334 (4.2)	
Local anaesthesia ± sedation	106 (8.3)	161 (3.6)	64 (3.0)	331 (4.2)	
Not indicated	7 (0.5)	317 (7.1)	76 (3.6))	400 (5.1)	

* Represents p-values of comparison of gender, age, level of training of surgical and anaesthesia provider and type of anaesthesia between GH, RRH and the NRH respectively.

For peer review only

For peer review only - http://bmjopen.bmj.com/site/about/guidelines.xhtml

Table 3. Indications for surgical procedures for children in the public healthcare sector in Uganda 2013-2014

	General Hospital 1276 patients, n (%)	Regional Referral Hospital 4475 patients, n (%)	National Referral Hospital 2135 patients, n (%)	Total 7886 patients, n (%)
**Abdominal and colorectal symptoms and conditions (n= 3774)**				
Groin hernia and hydrocoele	499 (39.1)	650 (14.5)	120 (5.6)	1269 (16.1)
Peritonitis	49 (3.8)	318 (7.1)	106 (5.0)	473 (6.0)
Umbilical hernia	113 (8.9)	185 (4.1)	72 (3.4)	370 (4.7)
Gut perforation	31 (2.4)	240 (5.4)	61 (2.9)	332 (4.2)
Imperforate anus	13 (1.0)	109 (2.4)	163 (7.6)	285 (3.6)
Intussusception	18 (1.4)	130 (2.9)	126 (5.9)	274 (3.5)
Intestinal Obstruction	47 (3.7)	124 (2.8)	89 (4.2)	260 (3.2)
Appendicitis	24 (1.9)	60 (1.3)	42 (2.0)	126 (1.6)
Abdominal injuries (blunt & penetrating)	10 (0.8)	62 (1.4)	37 (1.7)	109 (1.4)
Ruptured spleen	12 (0.9)	60 (1.3)	18 (0.8)	90 (1.1)
Colostomy/ closure of colostomy	10 (0.8)	44 (1.0)	20 (0.9)	74 (1.0)
Other	19 (1.5)	74 (1.7)	19 (0.9)	112 (1.4)
**Orthopaedic conditions (n=1264)**				
Orthopaedic trauma	62 (4.9)	366 (8.2)	224 (10.5)	652 (8.2)
Chronic osteomyelitis and septic arthritis	137 (10.7)	254 (5.7)	82 (3.8)	473 (6.0)
Club foot	61 (4.8)	53 (1.2)	25 (1.2)	139 (1.8)
Total	260 (20.4)	673 (15.0)	331 (15.5)	1264 (16.0)
**Ear, Nose, Throat conditions (n=721)**				
Tonsillitis/enlarged adenoids/ Foreign body, goitre	1 (0)	548 (12.2)	172 (8.0)	721 (9.1)
**Urology (n=444)**				
Cryptochidism	30 (2.4)	63 (1.4)	12 (0.6)	105 (1.3)
Testicular torsion	8 (0.6)	41 (0.9)	36 (1.7)	85 (1.1)
Other	7 (0.5)	138 (3.1)	107 (5.0)	251 (3.2)
**Neurosurgical conditions (n=316)**				
Spina bifida, hydrocephalus, Meningocoele, subdural haemorrhage, trauma	0 (0.0)	135 (3.0)	181 (8.5)	316 (4.0)
**Congenital heart disease (n=125)**	0 (0.0)	3 (0.1)	122 (5.7)	125 (1.6)
**Plastic surgical conditions (n=503)**				
Cleft lip and palate	27 (2.1)	107 (2.4)	10 (0.5)	144 (1.8)
Burns (acute and secondary surgery)	50 (3.9)	243 (5.4)	66 (3.1)	359 (4.6)
**Malignancies (n=92)**	1 (0.1)	68 (1.5)	24 (1.1)	92 (1.1)
**Other*, (n=896)**	67 (5.3)	630 (14.1)	212 (9.9)	896 (11.4)
**Not indicated (n=51)**	7 (0.5)	36 (0.8)	8 (0.4)	51 (0.6)

* Indications with ≤ 1%, were grouped together under “other”. Some patients had more than one indication for surgery, hence total number of conditions and symptoms exceeds number of patients.

For peer review only - http://bmjopen.bmj.com/site/about/guidelines.xhtml

Table 4. Paediatric surgical procedures by hospital level in Uganda 2013-2014.

**Procedure**	**General Hospital, 1276 patients, n (%)**	**Regional Referral Hospital, 4475 patients, n (%)**	**National Referral Hospital, 2135 patients, n (%)**	**Total n=7886 patients, n (%)**
**Abdominal and colorectal surgery (n=4559 procedures)**				
Laparotomy	179 (14.0)	990 (22.1)	453 (21.2)	1622 (20.6)
Herniotomy/herniorrhaphy/hydrocelectomy	591 (46.3)	652 (14.6)	121 (5.7)	1364 (17.3)
Colostomy (creating/closure)	17 (1.3)	206 (4.6)	175 (8.2)	398 (5.0)
Mayo's repair of umbilical hernia	113 (8.9)	179 (4.0)	68 (3.2)	360 (4.6)
Resection and anastomosis	12 (0.9)	149 (3.3)	83 (3.9)	244 (3.1)
Appendicectomy	30 (2.4)	58 (0.8)	45 (2.1)	133 (1.7)
Splenectomy	7 (0.5)	73 (1.6)	13 (0.6)	93 (1.2)
Other abdominal procedures (Swenson pull through, posterior sagittal anorectoplasty, Ramtsaedt's procedure.)	0 (0.0)	195 (4.4)	150 (7.0)	345 (4.4)
**Orthopaedic procedures (n=1212 procedures)**				
Fracture surgery (internal and external fixations), amputations.	72 (5.6)	452 (10.1)	154 (7.2)	678 (8.6)
Sequestrectomy	115 (9.0)	141 (3.2)	54 (2.5)	310 (3.9)
Corrective procedures for club foot	61 (4.8)	63 (1.4)	25 (1.2)	149 (1.9)
Arthrotomy	9 (0.7)	43 (1.0)	23 (1.1)	75 (1.0)
**Ear Nose Throat (n=594 procedures)**				
Adenotonsillectomy	0 (0)	292 (6.5)	115 (5.4)	407 (5.2)
FB removal, bronchoscopy, others	2 (0.2)	134 (3.0)	51 (2.4)	187 (2.4)
**Urology (n=429)**				
Orchidopexy and orchidectomy	41 (3.2)	115 (2.6)	34 (1.6)	190 (2.4)
Reconstructive surgery (hypospadia, epispadia, urethral valve)	4 (0.3)	130 (3.0)	105 (5.0)	239 (3.0)
**Cardiothoracic and vascular surgery (n=128 procedures)**	0 (0)	0 (0)	128 (6.0)	128 (1.6)
**Plastic Surgery (n=293 procedures)**				
Cleft lip and palate	31 (2.4)	132 (2.9)	50 (2.3)	213 (2.7)
Skin grafting	22 (1.7)	43 (1.0)	15 (0.7)	80 (1.0)
**Neurosurgery (n=285 procedures)**				
Neurosurgery procedures (VP shunt, Drainage of brain abscess, craniotomy, Repair of meninges	0 (0)	122 (2.7)	163 (7.6)	285 (3.6)
**Other procedures (n=782 procedures)***	92 (7.2)	575 (12.8)	115 (5.4)	782 (9.9)
**Not indicated (n=16 procedures)**	0 (0)	16 (0.4)	0 (0)	16 (0.2)

* Procedures with ≤ 1%, were grouped under "other". Some patients had more than one surgical procedure performed, hence total number of procedures exceeds number of patients.

For peer review only - http://bmjopen.bmj.com/site/about/guidelines.xhtml

Figure legend Figure 1. Study Hospitals.

Northern region: 7,188,139 inhabitants, (Apac GH, Nebbi GH, Kitgum GH, Matany GH, Lira RRH, Gulu RRH, and Moroto RRH). **Eastern region:** 9,042,422 inhabitants, (Lwala GH, Tororo GH, Iganga GH, Soroti RRH, Mbale RRH, Jinja RRH). **Western region:** 8,874,862 inhabitants, (Kilembe GH, Kisoro GH, Masindi GH, Itojo GH, Hoima RRH, Fort portal RRH, Mbarara RRH/University teaching hospital. **Central region:** 9,529,227 inhabitants. (Entebbe GH, Kalizizo GH, Mityana GH, Masaka RRH, Naguru RRH, Mubende RRH, Mulago NRH/Makerere University teaching hospital).

Uganda Bureau of Statistics. National Population and Housing Census 2014. https://www.ubos.org/wpcontent/ uploads/publications/03_20182014_National_Census_Main_Report.pdf.Total pop 34.6 million 0-17 yrs.=55.1%

For peer review only

For peer review only - http://bmjopen.bmj.com/site/about/guidelines.xhtml

Figure 1. Volumes and rates of paediatric surgery per region in Uganda.

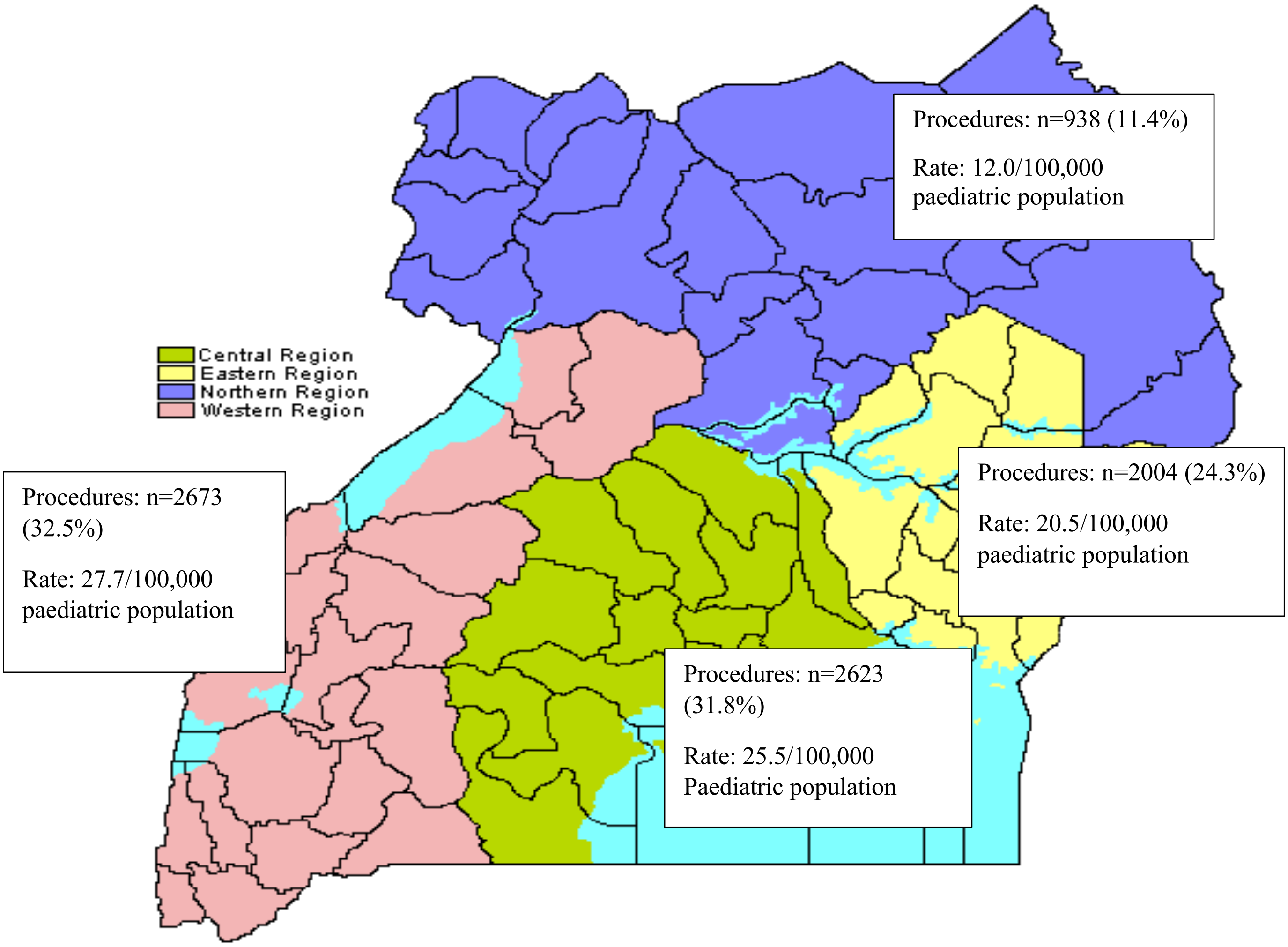

Northern region: 7,188,139 inhabitants, (Apac GH, Nebbi GH, Kitgum GH, Matany GH, Lira RRH, Gulu RRH, and Moroto RRH). **Eastern region**: 9,042,422 inhabitants, (Lwala GH, Tororo GH, Iganga GH, Soroti RRH, Mbale RRH, Jinja RRH). **Western region**: 8,874,862 inhabitants, (Kilembe GH, Kisoro GH, Masindi GH, Itojo GH, Hoima RRH, Fort portal RRH, Mbarara RRH/University teaching hospital. **Central region**: 9,529,227 inhabitants. (Entebbe GH, Kalizizo GH, Mityana GH, Masaka RRH, Naguru RRH, Mubende RRH, Mulago NRH/Makerere University teaching hospital).

only

Uganda Bureau of Statistics. National Population and Housing Census 2014. https://www.ubos.org/wp-content/uploads/publications/03_20182014_National_Census_Main_Report.pdf.Total pop 34.6 million 0-17 yrs.=55.1%

Appendix 1. Age distribution by hospital level.

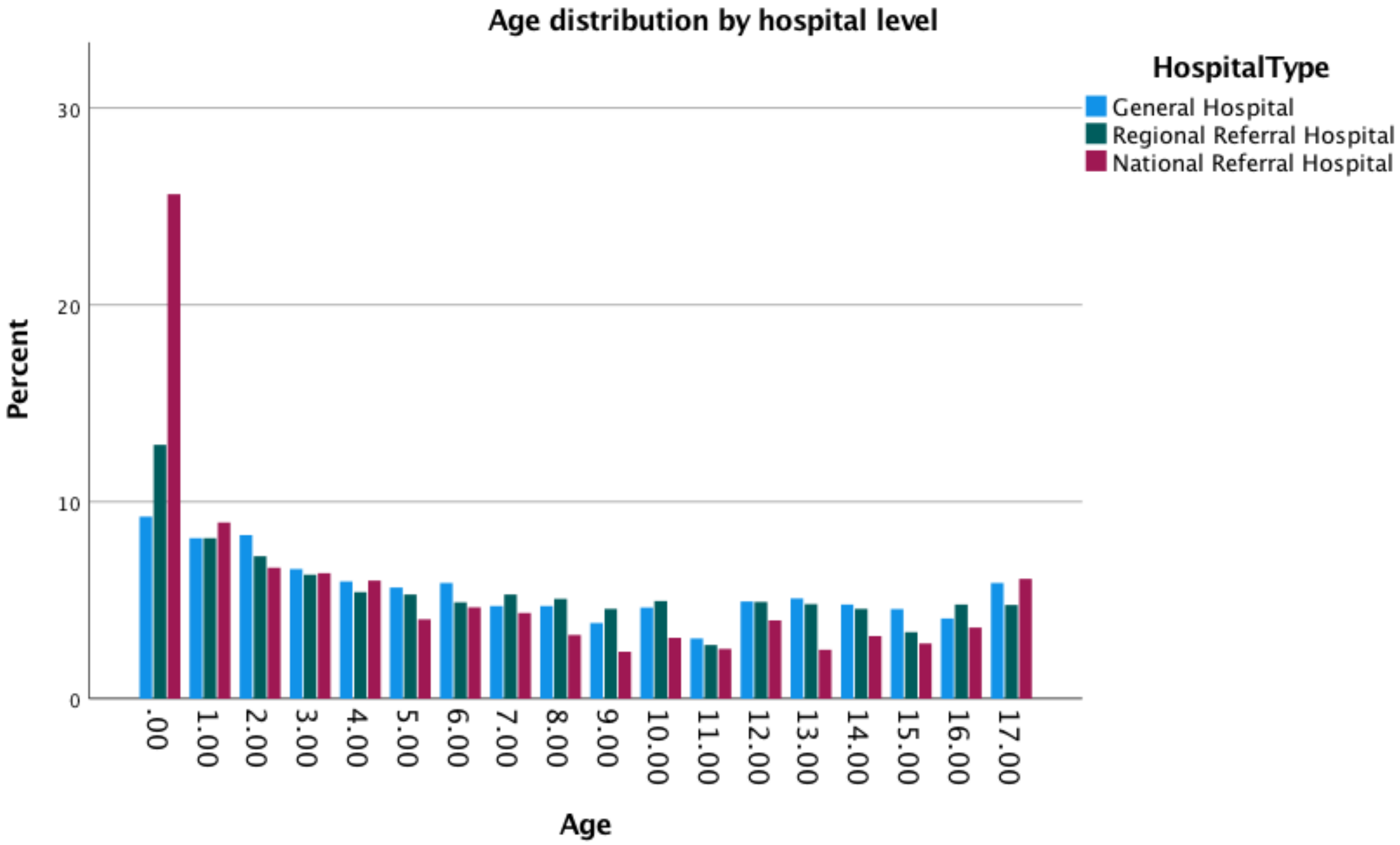

review only

BMJ Open

Surgical procedures for children in the public health care sector – a nationwide, facility-based study in Uganda

Journal:	*BMJ Open*
Manuscript ID	bmjopen-2020-048540.R2
Article Type:	Original research
Date Submitted by the Author:	18-Jun-2021
Complete List of Authors:	Ajiko, Mary Margaret; Soroti Regional Referral Hospital, Surgery<br>kressner, Julia; 1. Department of molecular medicine and Surgery, Karolinska Institute,<br>Matovu, Alphonsus; Mubende Regional Referral Hospital, Surgery; Karolinska Institute, Molecular Medicine and Surgery<br>Nordin, P.; Umeå University, Department of Surgical and Perioperative Sciences; Östersunds sjukhus, department of surgery<br>Wladis, Andreas; Linköping university, Department of Clinical and Experimental Medicine<br>Löfgren, Jenny; Karolinska Institute, Molecular Medicine and Surgery; Karolinska University Hospital, Reconstructive Plastic Surgery
**Primary Subject Heading**:	Surgery
Secondary Subject Heading:	Global health, Anaesthesia, Paediatrics, Health services research
Keywords:	SURGERY, PUBLIC HEALTH, PAEDIATRIC SURGERY

SCHOLARONE™
Manuscripts

I, the Submitting Author has the right to grant and does grant on behalf of all authors of the Work (as defined in the below author licence), an exclusive licence and/or a non-exclusive licence for contributions from authors who are: i) UK Crown employees; ii) where BMJ has agreed a CC-BY licence shall apply, and/or iii) in accordance with the terms applicable for US Federal Government officers or employees acting as part of their official duties; on a worldwide, perpetual, irrevocable, royalty-free basis to BMJ Publishing Group Ltd ("BMJ") its licensees and where the relevant Journal is co-owned by BMJ to the co-owners of the Journal, to publish the Work in this journal and any other BMJ products and to exploit all rights, as set out in our licence.

The Submitting Author accepts and understands that any supply made under these terms is made by BMJ to the Submitting Author unless you are acting as an employee on behalf of your employer or a postgraduate student of an affiliated institution which is paying any applicable article publishing charge ("APC") for Open Access articles. Where the Submitting Author wishes to make the Work available on an Open Access basis (and intends to pay the relevant APC), the terms of reuse of such Open Access shall be governed by a Creative Commons licence – details of these licences and which Creative Commons licence will apply to this Work are set out in our licence referred to above.

Other than as permitted in any relevant BMJ Author's Self Archiving Policies, I confirm this Work has not been accepted for publication elsewhere, is not being considered for publication elsewhere and does not duplicate material already published. I confirm all authors consent to publication of this Work and authorise the granting of this licence.

For peer review only

Surgical procedures for children in the public health care sector – a nationwide, facility-based study in Uganda

Mary Margaret Ajiko (MD)[1,2], Julia Kressner (MD)[1], Alphonsus Matovu (MD)[3], Pär Nordin (PhD)[4], Andreas Wladis (PhD)[5], Jenny Löfgren (PhD)[1]

1. Department of Molecular Medicine and Surgery, Karolinska Institute, Sweden
2. Soroti Regional Referral Hospital, Uganda
3. Mubende Regional Referral Hospital, Uganda
4. Department of Surgery and Perioperative Sciences, Umeå University, Sweden
5. Department of Clinical and Experimental Medicine, Linköping University, Sweden

Corresponding author

Dr Jenny Löfgren,

1. Department of Molecular Medicine and Surgery, Karolinska Institutet.

Karolinska University Hospital L1:00. 17176 Stockholm, Sweden.

2. Karolinska University Hospital, Reconstructive Plastic Surgery

Stockholm, SE 171 76

e-mail: jenny.lofgren@ki.se

Word Count: 3207

For peer review only - http://bmjopen.bmj.com/site/about/guidelines.xhtml

Abstract

Objective

This study investigated the surgical services for children at the highest levels of the public healthcare sector in Uganda. The aim was to determine volumes and types of procedure performed, the patients and the human resource involved.

Design

The study was a facility-based, record review.

Setting

The study was carried out at the National Referral Hospital, all 14 regional referral hospitals and 14 general hospitals in Uganda, representing the highest levels of hospital in the public healthcare sector.

Participants

The subjects were children <18 years who underwent major surgery in the study hospitals during 2013 and 2014.

Results

The study hospitals contribute with an average annual rate of paediatric surgery at 22.0 per 100,000 paediatric population. This is a fraction of the estimated need. Most of the procedures were performed for congenital anomalies (n=3111, 39.4%), inflammation and infection (n=2264, 28.7%), and trauma (n=1210, 15.3%). Specialist surgeons performed

For peer review only - http://bmjopen.bmj.com/site/about/guidelines.xhtml

60.3% (n=4758) of the procedures and anaesthesia was administered by specialist physician anaesthetists in 11.6% (n=917) of the cases.

Conclusions

A variety of paediatric surgical procedures are performed in a relatively decentralised system throughout Uganda. Task shifting and task sharing of surgery and anaesthesia are widespread: a large proportion of surgical procedures were carried out by non-specialist physicians, with anaesthesia mostly delivered by non-physician anaesthetists. Reinforcing the capacity and promoting the expansion of the health facilities studied, in particular the general hospitals and regional referral hospitals, could help reduce the immense unmet need for surgical services for children in Uganda.

For peer review only

Article summary

Strengths and limitations of this study

- This study included information about surgical procedures carried out in children in 29 hospitals (primary, secondary and tertiary institutions) within the public health care sector all over Uganda, and therefore adds important information compared to previous research carried out in fewer hospitals or in a smaller geographical area.
- The names of the surgical and anaesthesia providers were captured in the data collection and this made it possible to find out to what extent task sharing in surgery and anaesthesia for paediatric patients is practised in Uganda.
- The main limitation of the study is the retrospective study design and that the level of detail was limited to that of the logbooks reviewed.
- The data collected is now (2021) 7-8 years old, preceding recent global surgery efforts.
- The study focuses on volumes of surgery and lacks outcomes data which is a limitation as quantity and quality of surgical services should ideally not be separated.

For peer review only

Introduction

In recent years, global surgery has become a field of its own with the goal of providing access to safe and affordable surgery for all. In 2017, 1.7 billion children and adolescents lacked access to surgical care (1). Many childhood surgical conditions can be treated successfully with surgery, while failure to do so may lead to lifelong complications, disability, or even death (2)(3). The provision of surgical services for children is highly cost-effective (4).

Children requiring surgical intervention make up a significant proportion of child admissions. Information available suggests that 6-12% of all paediatric admissions in sub-Saharan Africa are surgical, although the proportion may be higher in some urban areas (5). In a regional referral hospital in Uganda, a third of the patients admitted to a surgical department during a three-year period were below 18 years of age (6). This however is not enough to meet the need for surgical services for children. In a cross-sectional survey performed in Uganda, Sierra Leone, Rwanda and Nepal, 19% of children had a surgical condition and 62% of these children had at least one unmet surgical need (3). A study in Uganda found that 7.4% of children were currently suffering from an untreated surgical condition (7).

For peer review only

Uganda is a sub-Saharan, low-income country with a population of over 20 million children (8). With a birth rate of 5.0 children per women, the paediatric population is rapidly increasing (9). Although 80% of the Ugandan population live in rural areas, 95% of surgeons practice in an urban environment(10). The extent to which the healthcare system can provide surgical services for children at national level is not known. The objective of the study was to investigate volumes and types of paediatric surgery performed at the highest-level hospitals

For peer review only - http://bmjopen.bmj.com/site/about/guidelines.xhtml

in Uganda, to relate that to the need for surgery in the paediatric population and to describe the human resource delivering the services.

Method

Study design

This was a retrospective review of relevant hospital records.

Study location, study population and study size

Some 29 hospitals geographically distributed throughout Uganda were included. These hospitals represent the highest level of public health care and include the National Referral Hospital (Mulago), all 14 regional referral hospitals (RRH) and an additional 14 general hospitals (GH) (Figure 1). Data from two RRH were incomplete and they were therefore excluded from the analysis.

The public healthcare system in Uganda is divided into seven levels: from village health teams (level I) to health centres (levels II-IV) and hospitals (levels V-VII). The hospitals are divided into general (district) hospitals (GH, level V), regional referral hospitals (RRH, level VI) and the National Referral Hospital (NRH, level VII). All private hospitals, are classified as level V hospitals. Uganda has 113 general hospitals, 38 of which are public-service hospitals. The rest are private and private non-profit hospitals (11). Each RRH has at least one general hospital in its catchment area. Due to scarcity of suitable public general hospitals in the northern and eastern regions, two NGO general hospitals were included in the study.

For peer review only

The catchment populations between the GHs, RRHs and NRH overlap as they are meant to provide services of increasing complexity. The general hospitals and the regional referral hospitals were designed for a catchment population of 500,000 people and two million people respectively, but these hospitals cover much larger populations now due to rapid population growth. GHs have specialists in internal medicine, gynaecology and obstetrics and in paediatrics, but not in surgery. Non-specialist medical doctors perform surgical procedures. Each RRH has a mandate to receive referred patients from the regions they serve, but self-referral from other regions is also permitted. The RRHs employ general surgeons. The National Referral Hospital covers the entire population for highly specialised services. It is also a teaching facility of Makerere College of Health Sciences. The three paediatric surgeons and the paediatric anaesthetist in Uganda are employed at this institution.

Since 2001, all public healthcare facilities have been officially free of charge (12), which is why the public healthcare sector was selected for this survey. To describe the surgical activity for children here, the NRH and all the 14 RRHs were included. In addition, one general hospital per RRH was selected. It was hypothesized that the surgical activity for children in the GHs would be low, as no surgeons are employed in these hospitals. This selection of hospitals allowed comparisons between the three highest levels in the public health care sector.

Logbooks from operating theatres for the years 2013 and 2014 at the hospitals studied were photographed and the information extracted was entered into excel spreadsheets. Data on all major surgical procedures performed on children were included. The logbooks provided, for each hospital, information about children that had undergone a surgical procedure. They included date of procedure, age of the patient, diagnosis, type of surgical procedure

For peer review only - http://bmjopen.bmj.com/site/about/guidelines.xhtml

performed, names of surgeon and anaesthetist, and type of anaesthesia used. Each surgeon's and anaesthetist's training level was thereafter clarified with each hospital. In this study, a child is defined as "a human being below the age of 18 years", according to the United Nations Convention on Children's Rights. This is also in keeping with the Ugandan Constitution (13). In 2014, Uganda children 0-17 represented 55.1% of the population, or 19.1 million children (14).

A major surgical procedure was defined as "any procedure conducted in the operating theatre involving the incision, excision, manipulation or suturing of tissue which usually requires regional or general anaesthesia or profound sedation to control pain"(15). An anaesthetic assistant/officer was a healthcare provider who had undertaken a 2-3 year course in anaesthesia at a recognised institution, and had been certified by the Allied Health Professionals Council of Uganda (16). A senior house officer (SHO) was a resident in surgery or anaesthesia. An intern had completed medical school and formally practices under the supervision of a senior colleague. A specialist physician is a doctor who has completed advanced education and training in a specific field of medicine.

Data analysis

Data were analysed by region and by hospital type using standard descriptive methods. Data are presented to describe the basic characteristics of the patients, the procedures and the human resource providing the anaesthesia and surgical services. Analyses used for group comparisons were Pearson's *t* test for continuous data and the Chi square test for proportions and identification of associations between demographic characteristics and indications for surgery. We also categorised gender and age distribution with the number of procedures performed on children for each hospital level. Missing data on the number of procedures

For peer review only

performed was handled by multiple imputation. The surgical volumes at the NRH were distributed to the entire population with the relative proportion of 74% to the central region, 12% to the western region,13% to the eastern region and 1% to the northern region. This distribution was based on previous research on geographical distribution of paediatric surgical patients presenting to the NRH (17). The 2014 national population census for Uganda were used to calculate the rate of surgical procedures for children per 100,000 paediatric population for 2014. The paediatric population of Uganda for 2013 was calculated based on the 2014 national census and a population growth of 3.3% per year (18).

In order to relate the hospital surgical productivity to the need for surgery, a population-based study on paediatric surgical need carried out in Uganda in 2014 was used (7).

For identification of common diagnoses and procedures, only complete original data were used. Two regional referral hospitals were excluded from the analyses; one had no data for the entire study period and the other omitted patient age in the majority of cases, making it impossible to extract all paediatric cases.

Ethical considerations

Ethical approval was granted by the Makerere University School of Public Health (HDREC 076) and the Uganda National Council of Science and Technology (HS1892). The Medical Superintendent of each hospital approved collection of data from the theatre log books. The retrospective nature of the study meant that the treatment delivered to the patients was not affected. To protect their integrity, patients' names were not included.

For peer review only

Role of the funding source

The funder of the study had no role in the study design, data collection, data analysis, data interpretation, writing of the article or the decision to submit for publication. All authors had full access to all the data in the study and were responsible for the decision to submit for publication.

Patient and Public involvement

There was no involvement of patients or the public.

Results

A total of 7886 children who had undergone a major surgical procedure were identified in the theatre records of the 27 hospitals during the study period. After imputation for missing data, this number was 8238 of which 4680 were operated in 2013 and 3558 in 2014. This represented an average rate of 22.0 major surgical procedures per 100,000 paediatric population of Uganda in 2013-2014. The rate varied between the four regions in Uganda: from 12.0 per 100,000 children in the northern region to 27.7 per 100,000 children in the central region (Figure 1). There was considerable variation in surgical volumes within the RRH and GH groups and the most productive RRH performed 21 times more surgical procedures on children than the least productive.

A population-based study in Uganda previously investigated the prevalence of surgical conditions and the need for surgical consultation and surgical intervention among children in Uganda (7). In relation to the findings of this previous study, the study hospitals in the

For peer review only

present study contributed with surgical volumes corresponding to 4.9-7.6% of the need for major surgical procedures in children (Table 1).

The basic characteristics of the patients and the surgical and anaesthesia providers are shown in Table 2. Of the children, 43.4% (n=3422) were below five years of age, and 60.1% (n=4797) were boys. In Appendix 1, the age distribution by hospital level is visualised. Most of the procedures (n=4758, 60.3%) were performed by specialists in general surgery or specialists in other surgical specialities (paediatric surgery, gynaecology, orthopaedic surgery, cardiothoracic surgery, plastic surgery, neurosurgery, ear nose and throat surgery and urology). Overall, surgical residents performed 14.5% (n=1145) of the procedures and other non-specialist physicians performed 20.5% (n= 1616). Interns carried out 3.0% (n=237). Anaesthesia was rarely administered by a physician anaesthetist (n=917, 11.6%) or physician anaesthetic resident (n=818, 10.4%). The majority of the patients were anaesthetised by an anaesthetic assistant or anaesthetic officer (n=5469, 69.4%).

The surgical procedures were performed by 275 medical doctors with different levels of qualification: 103 specialist surgeons in different disciplines of whom three were paediatric surgeons, 57 resident surgeons in different surgical disciplines and 115 medical officers without specific training in surgery. Of the specialists, 40 performed surgery at the NRH and 61 at the RRH. Only two specialist surgeons performed surgical procedures at a GH. Anaesthesia was administered by 18 physician anaesthetists and 99 anaesthetic officers. Of the physician anaesthetists, three worked at a RRH and 15 at the NRH. No physician anaesthetist worked at a GH.

For peer review only - http://bmjopen.bmj.com/site/about/guidelines.xhtml

Indications for the surgical procedures are presented in Table 3. The most common were related to congenital anomalies (n=3111, 39.4%) infectious and inflammatory conditions (n=2264, 28.7%) and trauma (n=1210, 15.3%). Table 4 shows the surgical procedures that were carried out. Abdominal and colorectal surgical procedures were most common, followed by orthopaedic and ear, nose and throat (ENT) procedures. The types of procedure varied, and in the GH hernia surgery was the most common. Surgery for congenital heart conditions (n=128, 1.6%) was only performed at the NRH where there is a heart institute run by cardiothoracic surgeons. Neurosurgeons performed all neurosurgical procedures including ventriculoperitoneal shunts, spina bifida repair, brain abscess drainage, craniotomy and meningocele repair. Such procedures were carried out at the NRH (Mulago Hospital) and some RRHs.

Discussion

The study hospitals in the present study contributed with an average annual surgical rate of 22.0 major surgical procedures per 100,000 paediatric population in Uganda in 2013 and 2014. The patients were often below five years and the surgical procedures were mainly carried out for congenital anomalies, inflammatory and infectious conditions and trauma. Task sharing and task shifting were widely practised as non-specialists frequently performed surgical procedures and as non-physician clinicians administered anaesthesia in most of the cases.

A considerable variation in the number and rate of major procedures performed at the different hospitals, and within the four regions of the country, was seen. The northern region

For peer review only - http://bmjopen.bmj.com/site/about/guidelines.xhtml

was the most underserved, where hospitals are few and the distance to the national referral hospital is greatest. This is similar to the results of a previous study in Uganda, which found a positive correlation between burden of paediatric surgical disease and distance to the nearest healthcare unit offering surgery for children (19). The majority of the Ugandan population live in a rural setting with very limited financial resources (6)(20). Transport costs are likely to pose a barrier to surgical care for children there. This is confirmed by previous research in low- and middle-income countries showing that cost and travelling distance are barriers to surgical care access (21). Decentralised services are therefore needed.

The Global Initiative for Children's surgery developed the Optimal Resources for Children's Surgical Care, published in 2019. This document stratifies by level of hospital what services and human resource should be available at each level. The recommendations are in line with what is demonstrated in the present study. On GH level, intermediate surgical services, including hernia repair and acute abdominal and traumatic conditions, should be performed (22). In the general hospitals in the present study, hernia surgery represented the vast majority of procedures performed, followed by other abdominal and orthopaedic procedures. These procedures were carried out on all three levels but in addition, the RRHs and NRH performed procedures of increasing complexity including surgical subspecialities.

For essential surgical care to be accessible for all children in Uganda, service delivery in both GHs and RRHs should be further promoted. It is encouraging to see that surgery for children was delivered in these hospitals even if at rates that represent only a fraction of the need. The more productive hospitals in each group could serve as models for the less productive ones. A first goal would be for all hospitals on the same level to deliver the same services and similar volumes of surgery. The present study did not investigate the outcomes after surgery,

For peer review only - http://bmjopen.bmj.com/site/about/guidelines.xhtml

and this should be evaluated in future research. Volume and quality represent two sides of the same coin and capacity-building must take both into consideration.

Significantly more boys than girls were recorded in this study. Several of the most common diagnoses requiring surgery were gender-specific, or over-represented, in males. For example, inguinal hernia, which was the most common indication for surgery in the GHs, is more common in boys than in girls (23). Female-specific diagnoses such as gynaecological conditions were rare. This study could not determine whether boys have better access to surgery than girls, but future research into gender as a barrier to surgical care in children could investigate this further.

Task-sharing and task-shifting of surgical duties and anaesthesia delivery were routinely practiced in the study hospitals. Task sharing with medical doctors without specific training in surgery is common in many countries but the safety and effectiveness as well as the training available for this important group of surgical providers is not well understood (24). Ways in which the highest possible quality of paediatric surgical services can be achieved and maintained must be the subject of future research. Standardization of the surgical training, and support and monitoring of non-surgeons for the commonest surgical conditions should be prioritized. The anaesthetic officers in Uganda have formal training and their duties are probably better regulated than those of non-specialist medical doctors. Nonetheless, regular monitoring of the surgical and anaesthetic work forces and the institutions in which they work is an essential component of the provision of high-quality and cost-effective surgical services for children. An additional important role of the health care system is to promote prevention: many surgical conditions can be prevented. Antenatal care and

For peer review only - http://bmjopen.bmj.com/site/about/guidelines.xhtml

For peer review only

vaccinations are carried out in the study hospitals and health information and child safety issues could be discussed with parents during such visits.

Surgery has an important place in several of the health targets in the UN's Sustainable Development Goal 3 (25). Uganda has a national plan for service standards, and service delivery standards for the health sector include a package for surgical, obstetrics, anaesthesia plans in scaling up surgical volumes and access to surgical care. However, a defined paediatric surgical package is not included (26). To reduce the death toll and disability caused by surgical conditions in the paediatric population, the healthcare system must be improved in order to deliver safe surgery and prevention strategies. Advancing and promoting the service delivery already in place could be part of the solution to reach the goal of surgical services of quality for all children with surgical conditions.

Strengths and Limitations

This study set out to investigate the situation for surgery for children in the public healthcare system in Uganda and to describe patients and their surgical providers at the three highest levels of hospitals. The study has several limitations. Due to limited resources, not all hospitals in the country could be visited and therefore overall volumes and surgical rates could not be calculated. The hospitals studied were the healthcare facilities first in line for the implementation of new policies of intervention, where Ministry of Health policy as well as increased funding would have a direct effect. Our choice of hospitals was thus relevant regarding current capacity and surgical volume, providing baseline information that is essential in the future evaluation of the effects of investment and interventions made to enhance and expand paediatric surgical services.

For peer review only - http://bmjopen.bmj.com/site/about/guidelines.xhtml

Given the retrospective collection of data in this study, the level of detail is limited to that of the logbooks reviewed. Information on outcome after paediatric surgery in relation to level of hospital and level of training of the surgical and anaesthesia providers would be very valuable. Where data on volumes of surgery performed during the study period were missing, in the selected hospitals, these were imputed as the best option. However, the data sufficed to provide important information about the state of surgery for children in Uganda.

The present data were collected in 2015 - 2017 and since then no significant investments have been made for improving surgical services for children on national level. There is still (2021) only one paediatric anaesthetist, three registered paediatric surgeons, and two hospitals with dedicated paediatric operating theatres. The findings should thus still apply today.

Acknowledgements

The authors acknowledge the invaluable assistance of the hospital directors and staff which made the study possible. The authors would like to thank Anna Peterson, William Yu and Andreas Vikholm for their important contribution in data collection and management. We also thank Dr Matteo Bottai for statistical assistance.

Funding

This work was supported through a grant from the Department of Public Health and Clinical Medicine, Unit of Research, Education, and Development, Östersund, Umeå University and by the Swedish Research Council.

For peer review only - http://bmjopen.bmj.com/site/about/guidelines.xhtml

Data sharing

Data may be shared upon request to the corresponding author.

Conflict of competing interests

There are no personal conflicts of interest or financial conflicts of interest among the authors.

Authors' Contributions

Study conception and design:
Jenny Löfgren, Andreas Wladis, Ajiko Mary Margaret, Pär Nordin.

Data acquisition:
Ajiko Mary Margaret, Jenny Löfgren, Julia Kressner, Alphonsus Matovu

Analysis and data interpretation:
Ajiko Mary Margaret, Jenny Löfgren, Julia Kressner, Andreas Wladis, Pär Nordin, Alphonsus Matovu

Drafting of the manuscript:
Ajiko Mary Margaret

Critical revision,
Ajiko Mary Margaret, Jenny Löfgren, Andreas Wladis, Par Nordin, Alphonsus matovu, Julia Kressner.

References

1. Mullapudi B, Grabski D, Ameh E, Ozgediz D, Thangarajah H, Kling K, et al. Estimates of number of children and adolescents without access to surgical care. Bull World Health Organ. 2019 Apr 1;97(4):254–8.

2. Walker IA, Obua AD, Mouton F, Ttendo S, Wilson IH. Paediatric surgery and anaesthesia in south-western Uganda: a cross-sectional survey. Bull World Health Organ. 2010 Dec 1;88(12):897–906.

3. Bickler SW, Rode H. Surgical services for children in developing countries. Bull World Health Organ. 2002;80:829–35.

4. Saxton AT, Poenaru D, Ozgediz D, Ameh EA, Farmer D, Smith ER, et al. Economic Analysis of Children's Surgical Care in Low- and Middle-Income Countries: A Systematic Review and Analysis. PloS One. 2016;11(10):e0165480.

For peer review only - http://bmjopen.bmj.com/site/about/guidelines.xhtml

5. Linden AF, Sekidde FS, Galukande M, Knowlton LM, Chackungal S, McQueen KAK. Challenges of surgery in developing countries: a survey of surgical and anesthesia capacity in Uganda's public hospitals. World J Surg. 2012 May;36(5):1056–65.

6. Tumusiime G, Was A, Preston MA, Riesel JN, Ttendo SS, Firth PG. The Quality and Utility of Surgical and Anesthetic Data at a Ugandan Regional Referral Hospital. World J Surg. 2017 Feb;41(2):370–9.

7. Butler EK, Tran TM, Fuller AT, Brammell A, Vissoci JR, de Andrade L, et al. Quantifying the pediatric surgical need in Uganda: results of a nationwide cross-sectional, household survey. Pediatr Surg Int. 2016;32(11):1075–85.

8. Population ages 0-14, total - Uganda | Data [Internet]. [cited 2020 Dec 22]. Available from: https://data.worldbank.org/indicator/SP.POP.0014.TO?locations=UG

9. Fertility rate, total (births per woman) - Uganda | Data [Internet]. [cited 2020 Dec 22]. Available from: https://data.worldbank.org/indicator/SP.DYN.TFRT.IN?locations=UG

10. Andersson O, Radell P, Ringo V, Mulungu M, Baker T. Quality of pediatric anesthesia: A cross-sectional study of a university hospital in a low-income country. PloS One. 2018;13(4):e0194622.

11. Kisa P, Grabski DF, Ozgediz D, Ajiko M, Aspide R, Baird R, et al. Unifying children's surgery and anesthesia stakeholders across institutions and clinical disciplines: challenges and solutions from Uganda. World J Surg. 2019;1–15.

12. Uganda Ministry of Health. Health Sector Strategic Plan 2000/01 - 2004/05 [Internet]. Available from: https://afri-can.org/wp-content/uploads/2016/04/HSSP-2000-01.pdf

13. Children Act. In: Ugandan Constitution. 1997.

14. Uganda Bureau of Statistics. National Population and Housing Census 2014 - Main Report [Internet]. [cited 2018 Jun 9]. Available from: http://www.ubos.org/onlinefiles/uploads/ubos/NPHC/NPHC%202014%20FINAL%20RESULTS%20REPORT.pdf

15. World Health Organization. Safe surgery saves lives. In: Second global patient safety challenge. 2008. p. 4.

16. Allied Health Professionals Council. The Allied Health Professionals Act - Chapter 268. In: The Allied Health Professionals Act.

17. Ullrich SJ, Kakembo N, Grabski DF, Cheung M, Kisa P, Nabukenya M, et al. Burden and Outcomes of Neonatal Surgery in Uganda: Results of a Five-Year Prospective Study. J Surg Res. 2020;246:93–9.

18. Population growth (annual %) - Uganda | Data [Internet]. [cited 2020 Dec 22]. Available from: https://data.worldbank.org/indicator/SP.POP.GROW?locations=UG

For peer review only

For peer review only - http://bmjopen.bmj.com/site/about/guidelines.xhtml

19. Smith ER, Vissoci JRN, Rocha TAH, Tran TM, Fuller AT, Butler EK, et al. Geospatial analysis of unmet pediatric surgical need in Uganda. J Pediatr Surg. 2017;52(10):1691–8.

20. Poverty headcount ratio at USD3.10 a day (2011 PPP) (% of population) [Internet]. The World Bank; 2012. Available from: data.worldbank.org/indicator/SI.POV.NAHC?locations=UG

21. Grimes CE, Bowman KG, Dodgion CM, Lavy CBD. Systematic Review of Barriers to Surgical Care in Low-Income and Middle-Income Countries. World J Surg. 2011 May;35(5):941–50.

22. Optimal Resources for Children's surgery [Internet]. Global Initiative for Children's Surgery. [cited 2020 Dec 6]. Available from: https://www.globalchildrenssurgery.org/optimal-resources/

23. Burcharth J, Pedersen M, Bisgaard T, Pedersen C, Rosenberg J. Nationwide Prevalence of Groin Hernia Repair. PLOS ONE. 2013 Jan 14;8(1):e54367.

24. Falk R, Taylor R, Kornelsen J, Virk R. Surgical Task-Sharing to Non-specialist Physicians in Low-Resource Settings Globally: A Systematic Review of the Literature. World J Surg. 2020 May;44(5):1368–86.

25. Home — SDG Indicators [Internet]. [cited 2020 Aug 19]. Available from: https://unstats.un.org/sdgs/#

26. HEALTH SECTOR DEVELOPMENT PLAN 2015/16 - 2019/20. :110.

For peer review only

For peer review only - http://bmjopen.bmj.com/site/about/guidelines.xhtml

Table 1. Surgical need and contribution towards the met need by the study hospitals

	Need for surgical consultation	Need for surgical intervention	Children operated in study hospitals	Interpretation
**Proportion of children**	7.4% *	7.6% - 11.8% of children with need for surgical consultation *	4.9 - 7.6 % of estimated need for a major surgical procedure	The 8238 children operated correspond to 4.9-7.6% of the estimated need for a major surgical procedure in the paediatric population of Uganda in 2014
**Number of children**	1,413,400 *<br><br>(7.4% of 19.1 million paediatric population in 2014)	107,418 – 166,781 *	8238	Another 99,000 - 159,000 surgical procedures are needed to meet the need for major surgical interventions in children in Uganda
**Rate, n per 100,000 paediatric population**	7400 *	562 – 873 *	22.0	The surgical rate contributed by the study hospitals is a fraction of the estimated need.

*** Reference 7. Butler et al**

For peer review only - http://bmjopen.bmj.com/site/about/guidelines.xhtml

Table 2. Basic characteristics of patients and human resource performing surgery for children per hospital type

	General hospital, n=1276	Regional Referral Hospital, n=4475	National Referral Hospital, n=2135	Total n=7886	p-value
**Sex, n (%)**					
Male	945 (74.1)	2516 (56.2)	1336 (62.6)	4797 (60.8)	<0.001*
Female	324 (25.4)	1620 (36.2)	756 (35.4)	2700 (34.2)	
Not indicated	7 (0.5)	339 (7.6)	43 (2)	389 (5.0)	
**Age distribution (years)**					
Mean (SD)	7.4 (5.5)	7.1 (5.5)	5.8 (5.7)	5.3 (4.7)	<0.001*
<5	488 (38.2)	1790 (40.0)	1144 (53.6)	3422 (43.4)	
>5	777 (60.9)	2677 (59.8)	980 (45.9)	4434 (56.6)	
Not indicated	11 (0.9)	8 (0.2)	11 (0.5)	30 (0.4)	
**Level of training of surgical provider**					
Specialist surgeon (including 3 paediatric surgeons)	324 (25.4)	3063 (68.4)	1371 (64.2)	4758 (60.2)	<0.001*
Resident	0	437 (9.8)	708 (33.2)	1145 (14.5)	
Medical officer	904 (70.8)	700 (15.6)	12 (0.6)	1616 (20.5)	
Intern	0	232 (5.2)	5 (0.2)	237 (3.0)	
Not indicated	48 (3.8)	43 (1.0)	39 (1.8)	130 (1.6)	
**Level of training of Anaesthesia provider**					
Physician Anaesthetists, (one paediatric anaesthesiologist)	0	807 (18.0)	110 (12.0)	917 (11.6)	<0.001*
Residents in anaesthesia,	0	163 (3.6)	655 (30.7)	818 (10.4)	
Self/by surgeon local anaesthesia	47 (3.7)	162 (3.6)	10 (0.5)	219 (2.8)	
Anaesthetic assistant or Officer	1190 (93.3)	3132 (70)	1147(53.7)	5469 (69.4)	
Not indicated	39 (3.1)	211 (4.7)	213 (10)	463 (5.9)	
**Type of Anaesthesia**					
General anaesthesia	1097 (86.0)	3811 (85.2)	1913 (89.6)	6821 (86.5)	<0.001*
Spinal anaesthesia	66 (5.2)	186 (4.2)	82 (3.8)	334 (4.2)	
Local anaesthesia ± sedation	106 (8.3)	161 (3.6)	64 (3.0)	331 (4.2)	
Not indicated	7 (0.5)	317 (7.1)	76 (3.6))	400 (5.1)	

* Represents p-values of comparison of gender, age, level of training of surgical and anaesthesia provider and type of anaesthesia between GH, RRH and the NRH respectively.

For peer review only - http://bmjopen.bmj.com/site/about/guidelines.xhtml

Table 3. Indications for surgical procedures for children in the public healthcare sector in Uganda 2013-2014

	General Hospital 1276 patients, n (%)	Regional Referral Hospital 4475 patients, n (%)	National Referral Hospital 2135 patients, n (%)	Total 7886 patients, n (%)
**Abdominal and colorectal symptoms and conditions (n= 3774)**				
Groin hernia and hydrocoele	499 (39.1)	650 (14.5)	120 (5.6)	1269 (16.1)
Peritonitis	49 (3.8)	318 (7.1)	106 (5.0)	473 (6.0)
Umbilical hernia	113 (8.9)	185 (4.1)	72 (3.4)	370 (4.7)
Gut perforation	31 (2.4)	240 (5.4)	61 (2.9)	332 (4.2)
Imperforate anus	13 (1.0)	109 (2.4)	163 (7.6)	285 (3.6)
Intussusception	18 (1.4)	130 (2.9)	126 (5.9)	274 (3.5)
Intestinal Obstruction	47 (3.7)	124 (2.8)	89 (4.2)	260 (3.2)
Appendicitis	24 (1.9)	60 (1.3)	42 (2.0)	126 (1.6)
Abdominal injuries (blunt & penetrating)	10 (0.8)	62 (1.4)	37 (1.7)	109 (1.4)
Ruptured spleen	12 (0.9)	60 (1.3)	18 (0.8)	90 (1.1)
Colostomy/ closure of colostomy	10 (0.8)	44 (1.0)	20 (0.9)	74 (1.0)
Other	19 (1.5)	74 (1.7)	19 (0.9)	112 (1.4)
**Orthopaedic conditions (n=1264)**				
Orthopaedic trauma	62 (4.9)	366 (8.2)	224 (10.5)	652 (8.2)
Chronic osteomyelitis and septic arthritis	137 (10.7)	254 (5.7)	82 (3.8)	473 (6.0)
Club foot	61 (4.8)	53 (1.2)	25 (1.2)	139 (1.8)
Total	260 (20.4)	673 (15.0)	331 (15.5)	1264 (16.0)
**Ear, Nose, Throat conditions (n=721)**				
Tonsillitis/enlarged adenoids/ Foreign body, goitre	1 (0)	548 (12.2)	172 (8.0)	721 (9.1)
**Urology (n=444)**				
Cryptochidism	30 (2.4)	63 (1.4)	12 (0.6)	105 (1.3)
Testicular torsion	8 (0.6)	41 (0.9)	36 (1.7)	85 (1.1)
Other	7 (0.5)	138 (3.1)	107 (5.0)	251 (3.2)
**Neurosurgical conditions (n=316)**				
Spina bifida, hydrocephalus, Meningocoele, subdural haemorrhage, trauma	0 (0.0)	135 (3.0)	181 (8.5)	316 (4.0)
**Congenital heart disease (n=122)**	0 (0.0)	0 (0.0)	122 (5.7)	122 (1.5)
**Plastic surgical conditions (n=503)**				
Cleft lip and palate	27 (2.1)	107 (2.4)	10 (0.5)	144 (1.8)
Burns (acute and secondary surgery)	50 (3.9)	243 (5.4)	66 (3.1)	359 (4.6)
**Malignancies (n=92)**	1 (0.1)	68 (1.5)	24 (1.1)	92 (1.1)
**Other*, (n=896)**	67 (5.3)	630 (14.1)	212 (9.9)	896 (11.4)
**Not indicated (n=51)**	7 (0.5)	36 (0.8)	8 (0.4)	51 (0.6)

* Indications with ≤ 1%, were grouped together under "other". Some patients had more than one indication for surgery, hence total number of conditions and symptoms exceeds number of patients.

For peer review only - http://bmjopen.bmj.com/site/about/guidelines.xhtml

Table 4. Paediatric surgical procedures by hospital level in Uganda 2013-2014.

Procedure	General Hospital, 1276 patients, n (%)	Regional Referral Hospital, 4475 patients, n (%)	National Referral Hospital, 2135 patients, n (%)	Total n=7886 patients, n (%)
**Abdominal and colorectal surgery (n=4559 procedures)**				
Laparotomy	179 (14.0)	990 (22.1)	453 (21.2)	1622 (20.6)
Herniotomy/herniorrhaphy/hydrocelectomy	591 (46.3)	652 (14.6)	121 (5.7)	1364 (17.3)
Colostomy (creating/closure)	17 (1.3)	206 (4.6)	175 (8.2)	398 (5.0)
Mayo's repair of umbilical hernia	113 (8.9)	179 (4.0)	68 (3.2)	360 (4.6)
Resection and anastomosis	12 (0.9)	149 (3.3)	83 (3.9)	244 (3.1)
Appendicectomy	30 (2.4)	58 (0.8)	45 (2.1)	133 (1.7)
Splenectomy	7 (0.5)	73 (1.6)	13 (0.6)	93 (1.2)
Other abdominal procedures (Swenson pull through, posterior sagittal anorectoplasty, Ramtsaedt's procedure.)	0 (0.0)	195 (4.4)	150 (7.0)	345 (4.4)
**Orthopaedic procedures (n=1212 procedures)**				
Fracture surgery (internal and external fixations), amputations.	72 (5.6)	452 (10.1)	154 (7.2)	678 (8.6)
Sequestrectomy	115 (9.0)	141 (3.2)	54 (2.5)	310 (3.9)
Corrective procedures for club foot	61 (4.8)	63 (1.4)	25 (1.2)	149 (1.9)
Arthrotomy	9 (0.7)	43 (1.0)	23 (1.1)	75 (1.0)
**Ear Nose Throat (n=594 procedures)**				
Adenotonsillectomy	0 (0)	292 (6.5)	115 (5.4)	407 (5.2)
FB removal, bronchoscopy, others	2 (0.2)	134 (3.0)	51 (2.4)	187 (2.4)
**Urology (n=429)**				
Orchidopexy and orchidectomy	41 (3.2)	115 (2.6)	34 (1.6)	190 (2.4)
Reconstructive surgery (hypospadia, epispadia, urethral valve)	4 (0.3)	130 (3.0)	105 (5.0)	239 (3.0)
**Cardiothoracic and vascular surgery (n=128 procedures)**	0 (0)	0 (0)	128 (6.0)	128 (1.6)
**Plastic Surgery (n=293 procedures)**				
Cleft lip and palate	31 (2.4)	132 (2.9)	50 (2.3)	213 (2.7)
Skin grafting	22 (1.7)	43 (1.0)	15 (0.7)	80 (1.0)
**Neurosurgery (n=285 procedures)**				
Neurosurgery procedures (VP shunt, Drainage of brain abscess, craniotomy, Repair of meninges	0 (0)	122 (2.7)	163 (7.6)	285 (3.6)
**Other procedures (n=782 procedures)***	92 (7.2)	575 (12.8)	115 (5.4)	782 (9.9)
**Not indicated (n=16 procedures)**	0 (0)	16 (0.4)	0 (0)	16 (0.2)

* Procedures with ≤ 1%, were grouped under "other". Some patients had more than one surgical procedure performed, hence total number of procedures exceeds number of patients.

For peer review only - http://bmjopen.bmj.com/site/about/guidelines.xhtml

Figure legend Figure 1. Volumes and rates of paediatric surgery per region in Uganda.

Northern region: 7,188,139 inhabitants, (Apac GH, Nebbi GH, Kitgum GH, Matany GH, Lira RRH, Gulu RRH, and Moroto RRH). **Eastern region:** 9,042,422 inhabitants, (Lwala GH, Tororo GH, Iganga GH, Soroti RRH, Mbale RRH, Jinja RRH). **Western region:** 8,874,862 inhabitants, (Kilembe GH, Kisoro GH, Masindi GH, Itojo GH, Hoima RRH, Fort portal RRH, Mbarara RRH/University teaching hospital. **Central region:** 9,529,227 inhabitants. (Entebbe GH, Kalizizo GH, Mityana GH, Masaka RRH, Naguru RRH, Mubende RRH, Mulago NRH/Makerere University teaching hospital).

Uganda Bureau of Statistics. National Population and Housing Census 2014. https://www.ubos.org/wpcontent/ uploads/publications/03_20182014_National_Census_Main_Report.pdf.Total pop 34.6 million 0-17 yrs.=55.1%

For peer review only

Figure 1. Volumes and rates of paediatric surgery per region in Uganda.

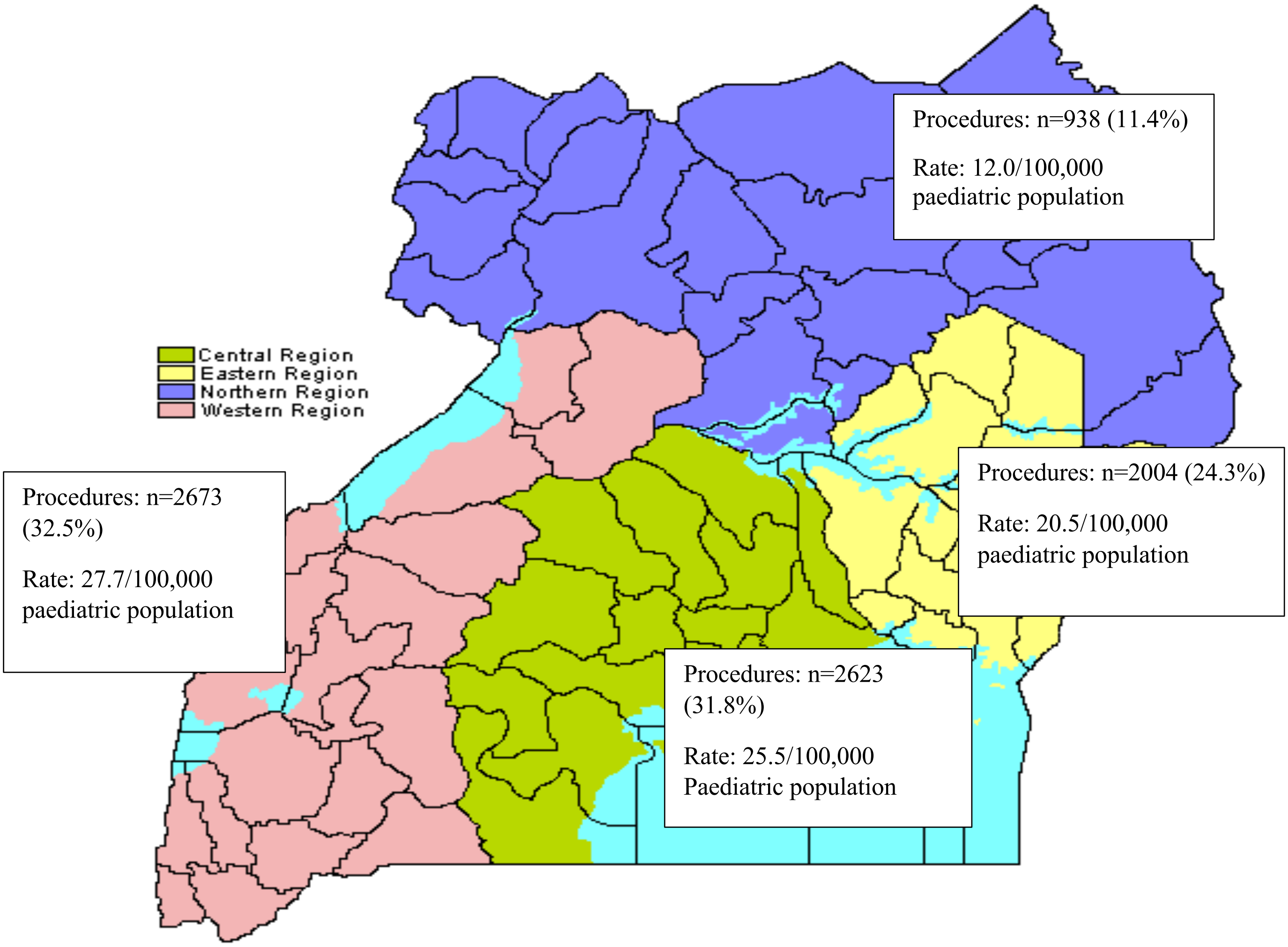

Northern region: 7,188,139 inhabitants, (Apac GH, Nebbi GH, Kitgum GH, Matany GH, Lira RRH, Gulu RRH, and Moroto RRH). **Eastern region**: 9,042,422 inhabitants, (Lwala GH, Tororo GH, Iganga GH, Soroti RRH, Mbale RRH, Jinja RRH). **Western region**: 8,874,862 inhabitants, (Kilembe GH, Kisoro GH, Masindi GH, Itojo GH, Hoima RRH, Fort portal RRH, Mbarara RRH/University teaching hospital. **Central region**: 9,529,227 inhabitants. (Entebbe GH, Kalizizo GH, Mityana GH, Masaka RRH, Naguru RRH, Mubende RRH, Mulago NRH/Makerere University teaching hospital).

Uganda Bureau of Statistics. National Population and Housing Census 2014. https://www.ubos.org/wp-content/uploads/publications/03_20182014_National_Census_Main_Report.pdf.Total pop 34.6 million 0-17 yrs.=55.1%

only

For peer review only - http://bmjopen.bmj.com/site/about/guidelines.xhtml

Appendix 1. Age distribution by hospital level.

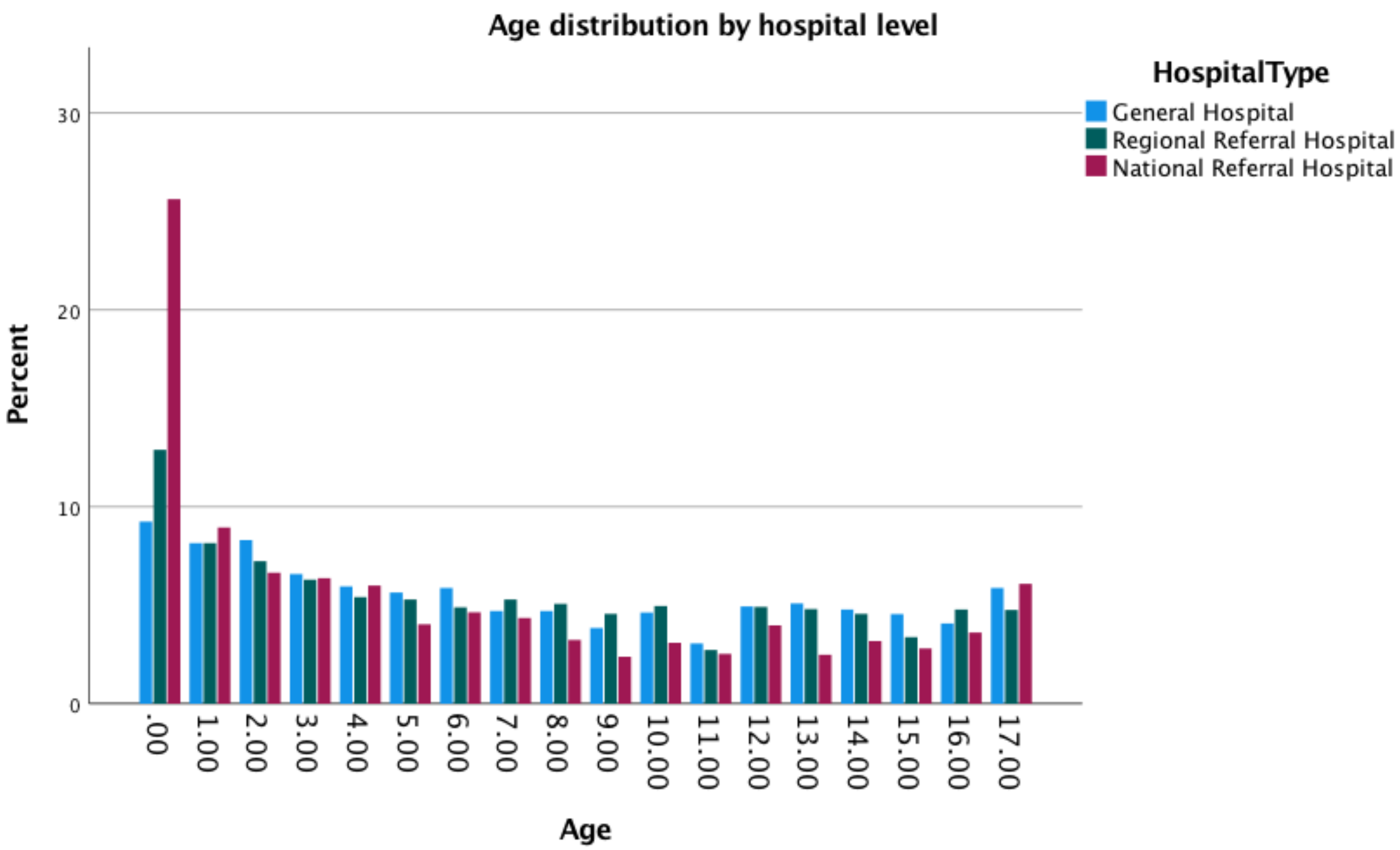

review only

STROBE Statement

Checklist of items that should be included in reports of observational studies

Section/Topic	Item No	Recommendation	Reported on Page No
**Title and abstract**	1	(*a*) Indicate the study's design with a commonly used term in the title or the abstract	1
		(*b*) Provide in the abstract an informative and balanced summary of what was done and what was found	2-3
**Introduction**			
Background/rationale	2	Explain the scientific background and rationale for the investigation being reported	5
Objectives	3	State specific objectives, including any prespecified hypotheses	5-6
**Methods**			
Study design	4	Present key elements of study design early in the paper	6
Setting	5	Describe the setting, locations, and relevant dates, including periods of recruitment, exposure, follow-up, and data collection	6-7
Participants	6	(*a*) *Cohort study*—Give the eligibility criteria, and the sources and methods of selection of participants. Describe methods of follow-up<br>*Case-control study*—Give the eligibility criteria, and the sources and methods of case ascertainment and control selection. Give the rationale for the choice of cases and controls<br>*Cross-sectional study*—Give the eligibility criteria, and the sources and methods of selection of participants	7-8
		(*b*) *Cohort study*—For matched studies, give matching criteria and number of exposed and unexposed<br>*Case-control study*—For matched studies, give matching criteria and the number of controls per case	
Variables	7	Clearly define all outcomes, exposures, predictors, potential confounders, and effect modifiers. Give diagnostic criteria, if applicable	7-8
Data sources/measurement	8*	For each variable of interest, give sources of data and details of methods of assessment (measurement). Describe comparability of assessment methods if there is more than one group	8-9
Bias	9	Describe any efforts to address potential sources of bias	8-9
Study size	10	Explain how the study size was arrived at	6
Quantitative variables	11	Explain how quantitative variables were handled in the analyses. If applicable, describe which groupings were chosen and why	8-9
Statistical methods	12	(*a*) Describe all statistical methods, including those used to control for confounding	8-9
		(*b*) Describe any methods used to examine subgroups and interactions	NA
		(*c*) Explain how missing data were addressed	8-9
		(*d*) *Cohort study*—If applicable, explain how loss to follow-up was addressed<br>*Case-control study*—If applicable, explain how matching of cases and controls was addressed<br>*Cross-sectional study*—If applicable, describe analytical methods taking account of sampling strategy	8-9
		(*e*) Describe any sensitivity analyses	NA

For peer review only

Section/Topic	Item No	Recommendation	Reported on Page No
**Results**			
Participants	13*	(a) Report numbers of individuals at each stage of study—eg numbers potentially eligible, examined for eligibility, confirmed eligible, included in the study, completing follow-up, and analysed	10
		(b) Give reasons for non-participation at each stage	
		(c) Consider use of a flow diagram	
Descriptive data	14*	(a) Give characteristics of study participants (eg demographic, clinical, social) and information on exposures and potential confounders	Table 2
		(b) Indicate number of participants with missing data for each variable of interest	Table 2-4
		(c) *Cohort study*—Summarise follow-up time (eg, average and total amount)	
Outcome data	15*	*Cohort study*—Report numbers of outcome events or summary measures over time	
		Case-control study—Report numbers in each exposure category, or summary measures of exposure	
		Cross-sectional study—Report numbers of outcome events or summary measures	Table 2-4
Main results	16	(*a*) Give unadjusted estimates and, if applicable, confounder-adjusted estimates and their precision (eg, 95% confidence interval). Make clear which confounders were adjusted for and why they were included	NA
		(*b*) Report category boundaries when continuous variables were categorized	NA
		(*c*) If relevant, consider translating estimates of relative risk into absolute risk for a meaningful time period	NA
Other analyses	17	Report other analyses done—eg analyses of subgroups and interactions, and sensitivity analyses	NA
**Discussion**			
Key results	18	Summarise key results with reference to study objectives	12
Limitations	19	Discuss limitations of the study, taking into account sources of potential bias or imprecision. Discuss both direction and magnitude of any potential bias	15-16
Interpretation	20	Give a cautious overall interpretation of results considering objectives, limitations, multiplicity of analyses, results from similar studies, and other relevant evidence	12-15
Generalisability	21	Discuss the generalisability (external validity) of the study results	16
**Other Information**			
Funding	22	Give the source of funding and the role of the funders for the present study and, if applicable, for the original study on which the present article is based	16

**Give information separately for cases and controls in case-control studies and, if applicable, for exposed and unexposed groups in cohort and cross-sectional studies.*

Note: An Explanation and Elaboration article discusses each checklist item and gives methodological background and published examples of transparent reporting. The STROBE checklist is best used in conjunction with this article (freely available on the Web sites of PLoS Medicine at http://www.plosmedicine.org/, Annals of Internal Medicine at http://www.annals.org/, and Epidemiology at http://www.epidem.com/). Information on the STROBE Initiative is available at www.strobe-statement.org.

For peer review only - http://bmjopen.bmj.com/site/about/guidelines.xhtml
